# Method for GHz optical helicity modulation by acoustic drum modes on a chip

**DOI:** 10.1038/s41598-025-26587-9

**Published:** 2025-11-07

**Authors:** N. Ashurbekov, I. dePedro-Embid, A. Pitanti, M. Msall, P. V. Santos

**Affiliations:** 1 Paul-Drude-Institut für Festkörperelektronik, Leibniz-Institut im Forschungsverbund Berlin e. V., Hausvogteiplatz 5-7, 10117 Berlin, Germany; 2 Dipartimento di Fisica E. Fermi, University of Pisa, largo Bruno Pontecorvo 3, 56127 Pisa, Italy; 3 Department of Physics and Astronomy, Bowdoin College, Brunswick, Maine 04011 USA

**Keywords:** Materials science, Optics and photonics, Physics

## Abstract

On-chip laterally confined GHz acoustic modes with tunable helicity open the way for advanced optomechanical functionalities. Here, we demonstrate a novel concept for the electrical generation of GHz membrane-like helical drum modes on a chip, and propose their application to the future generation of optical beams with tunable orbital angular momentum (OAM). Our concept relies on the strong dependence of the frequency spectrum of Lamb-like acoustic modes on the thickness of the propagating medium. Modes generated by a piezoelectric resonator in a thicker (or thinner) substrate region remain confined in this region via reflections at the lateral boundaries with thinner (thicker) substrate regions. If the generation region is disk-shaped, the lateral reflections form drum-like modes, which we experimentally confirm with radio-frequency spectroscopy and interferometric maps of the surface displacements. Furthermore, we show that an array of sector-shaped piezoelectric transducers powered with appropriate radio-frequency phases creates acoustic vortices with tunable OAM polarity, which we transfer to an optical beam. Our analytical and finite-element models yield useful insights into the acoustic coupling of bulk and surface modes that can guide adaptation to other material systems. These acoustic drum modes provide a flexible platform for acousto-optical chiral functionalities in the GHz frequency range.

## Introduction

Thin acoustic membranes form a powerful platform for sensing and transduction by combining a very small mass with record-high quality factors (Q)^1–3^. The high Q’s arise from the confinement of the vibrations induced by the large acoustic impedance mismatch with the clamping medium and enable large mechanical deformations with moderate external excitation. Both thin membranes and suspended beam structures can be conveniently defined using conventional lithography and under-etching procedures. These structures can be shaped into two-dimensional (2D) phononic and/or photonic crystals with enhanced opto-mechanical coupling. When combined with the large deformations, they become particularly interesting for ultra-sensitive sensing^4,5^ and the control of opto-^6–8^ and electronic quantum excitations^9–11^.

Recent demonstrations of membranes with topological phononic structures^12,13^ that support helical acoustic modes are exciting high interest for negative and asymmetric acoustic reflection^14^, non-reciprocal acoustic propagation^15^, the creation of synthetic gauge fields^16^, as well as the generation of chiral light^7^. However, the small thicknesses and masses of membrane structures, which ensure their high Q-factors, also limit the mechanical vibration frequencies. In a more general context, helical acoustic modes have been exploited for particle manipulation^17^ as well as for the acousto-optical generation of light beams with tunable orbital angular momentum (OAM)^17–20^ for applications in optical communication and advanced metrology^21^. With a few exceptions^20^, most of them have so far focused on acoustic modes with relatively low frequencies (i.e., well below a GHz) and, thus, response times.

**Fig. 1:**
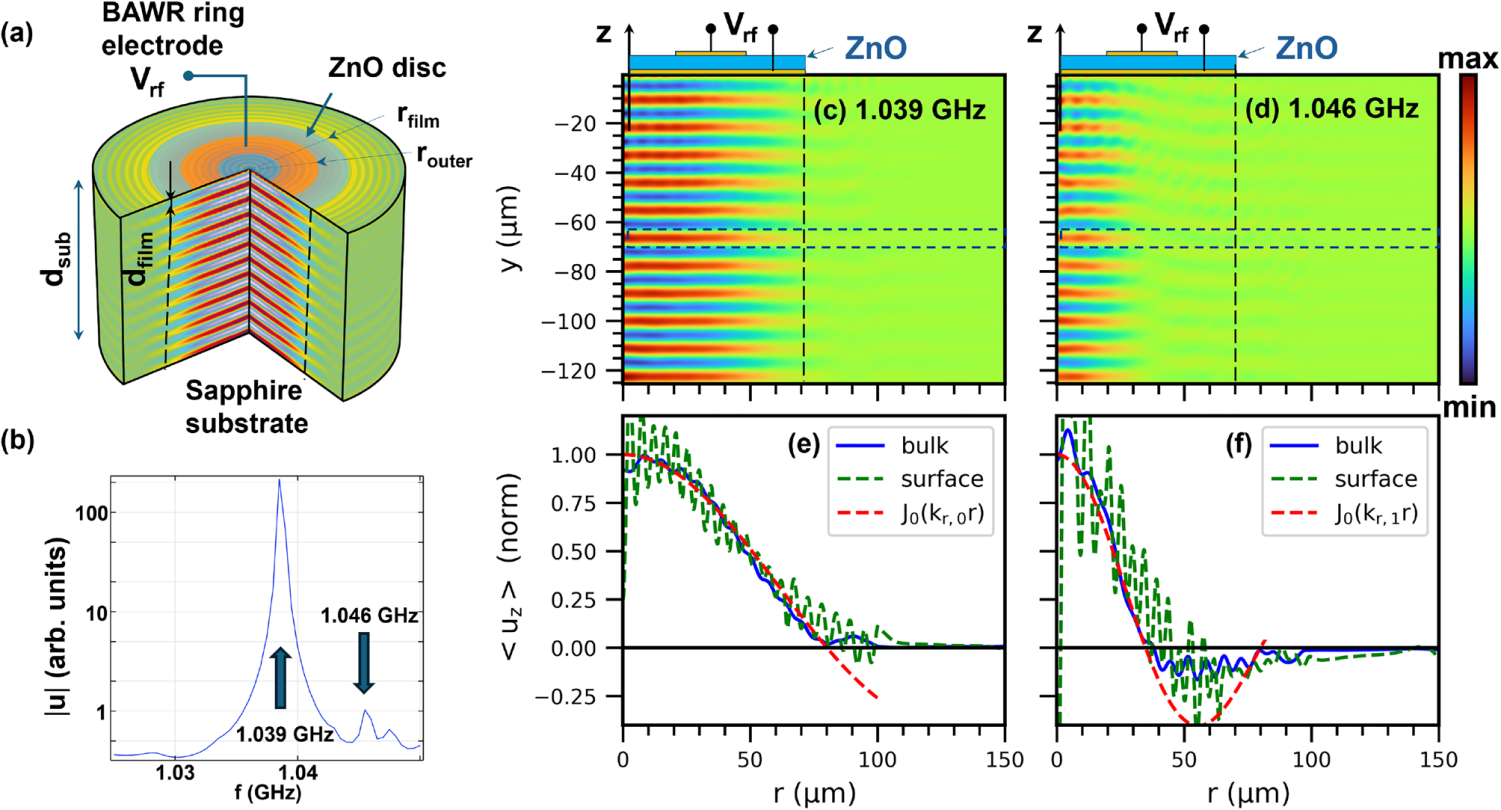
Laterally confined Lamb modes. (**a**) Finite-element (FEM) simulations of the acoustic displacement field ($\textbf{u}$) excited by a ring-shaped piezoelectric bulk acoustic wave resonator (BAWR) on a sapphire substrate (see “Methods” for detail). The BAWR consists of a piezoelectric ZnO layer of thickness $d_\textrm{film}=700$ nm shaped as a disk of radius of $r_\textrm{film}= 70~\upmu$m and sandwiched between two thin metal contacts (cf. upper part of panel **c**). The bottom contact is an Au ring with a radius $r_\textrm{outer}=27~\upmu$m while the top contact is a 30 nm-thick Al ring with inner and outer radii of $r_\textrm{inner}=21.5~\upmu$m and $r_\textrm{outer}=27~\upmu$m, respectively. The double-side polished c-plane sapphire substrate (thickness $d_\textrm{sub}=425~\upmu$m) forms Fabry–Pérot acoustic cavities with different lengths in the ZnO coated and uncoated regions, leading to different acoustic resonance frequencies in these two regions. The frequency mismatch laterally confines the acoustic modes in the regions underneath the ZnO-coated region, which is demarked by vertical dashed lines in panels (**a**), (**c**) and (**d**). (**b**) Calculated frequency ($f_\textrm{RF}$) spectrum of the average displacement amplitude $|\textbf{u}|$ excited by the radio-frequency (rf) voltage ($V_\textrm{rf}$) applied to the BAWR within a free spectral range of the acoustic cavity. (**c**,**d**) Vertical displacement field ($u_z$) for the two resonances indicated by the thick arrows in panel (**b**), corresponding to modes confined underneath the ZnO layers. (**e**,**f**) Radial dependence of the average acoustic amplitude $u_z$ integrated within the dashed rectangles (blue curves) and at the sample surface (green curves) in panels (**c**) and (**d**), respectively. The dashed red lines are Bessel-function fits to the profiles with different radial wavevectors $k_{r,m_r}$, ($m_r=1,2$) as discussed in “Radially confined lamb modes”. The material parameters used in the finite element simulation are listed in Table SMI of the Supplement (SM).

In this work, we demonstrate the piezoelectric excitation of membrane-like helical drum modes in the GHz range and their application for the generation of optical beams with tunable helicity.

Drum mode excitation takes place on a bulk substrate compatible with monolithic integration. The acoustic waves are electrically generated by a ring-shaped GHz bulk acoustic wave resonator (BAWR) consisting of two ring-shaped metal contacts sandwiching a larger, disk-shaped piezoelectric film. The finite radial dimension of the BAWR imparts a non-vanishing radial wave vector $k_r$ to the modes, which evolve into confined Lamb-like modes [to be denoted here simply as Lamb modes (propagating Lamb modes are, strictly speaking, modes propagating over distances much larger than the substrate thickness, which is not the case here.)] as they specularly reflect at the borders of the acoustic cavity formed in-between the (polished) top and bottom sample surfaces. We show that the slightly larger total thicknesses of the acoustic cavity underneath the disk-shaped piezoelectric film induces the confinement of the Lamb waves in this region, leading to the generation of drum modes with cylindrical symmetry.

Experimental evidence for the existence of drum modes in a similar structure was previously inferred from the radio-frequency (rf) spectroscopic response of a piezoelectric film coupled to a superconducting qubit^23^. Here, we directly prove their drum-like character by mapping the acoustic surface displacement with $\upmu$m lateral resolution using scanning optical interferometry. Furthermore, we demonstrate the excitation of GHz helical drum modes with tunable OAM polarity by an array of sector-shaped BAWRs powered with appropriate rf phases, which can be transferred to an optical beam. The interferometric phase shifts induced by the helical surface deformation are qualitatively similar to those from a reflective spiral phase plate^20,24^: reflection on this surface thus generates light with OAM that can be modulated at GHz frequencies. The optical generation of GHz helical acoustic phonons (see, eg., Ref. 25) as well as the electrical excitation of GHz modes with a fixed helical polarity employing spiral-like structures^20^ have recently been reported. The approach introduced here extends the control of the helical polarity to electrically generated phonons in the GHz range, a functionality so far missing for acousto-optical devices.

The experimental results are well-accounted for by finite-element and analytical models that yield useful insights into the acoustic coupling of bulk and surface modes and guide adaptation to other applications and material systems.

In the following sections, we first show the feasibility of generating GHz drum modes using BAWRs by presenting numerical simulations for the structure in Fig. 1a (“Radially confined Lamb modes”). The subsequent section first experimentally addresses the electrical response of the structures (“Electrical response”) as well as the lateral confinement of the acoustic modes (“Mapping of GHz drum modes”). We then proceed to the phase control of the helical polarity of the acoustic drum modes as well as its transfer to an optical beam (“Helical Drum Modes”). “Discussions” introduces an analytical model for the spatial distribution of the acoustic field, which provides insight into the nature of the observed modes and supports the conclusions summarized in “Conclusions”. This sections also addresses prospects for enhancing the acousto-optical coupling and the tunable helicity of the optical modes.

## Results

### Radially confined Lamb modes

Drum mode excitation on a bulk substrate is conceptually illustrated by the finite-element (FEM) simulations for the structure sketched in Fig. 1a (see “Methods” for details). Here, the ring-shaped orange area is the top BAWR metal contact and the gray disk the disk-shaped piezoelectric film [cf. Fig. 1a]. In our case, the latter consists of a textured ZnO piezoelectric film deposited on a c-plane sapphire substrate; the concept is, however, extendable to other material combinations. Due to its small ring width, the BAWR excites modes with a small radial wave vector $k_r$, which evolve into confined Lamb modes as they specularly reflect at the (polished) top and bottom sample surfaces. The volume between these surfaces forms Fabry–Pérot cavities for the Lamb modes with notably different resonance frequencies in the film-coated and uncoated areas. As a consequence, high-Q modes resonantly excited by the BAWR underneath the circular film do not normally find matching states in the surrounding area.

Despite the homogeneous substrate material, and similarly to membrane vibrations, the modes are reflected at the lateral film boundaries and remain confined within this region [demarked by vertical dashed lines in Fig. 1a], thus forming GHz drum-like modes with cylindrical symmetry, as indicated by the color-coded displacement amplitude shown in the figure. In contrast to other lateral confinement approaches developed in the BAWR technology^26^, the one presented here is intrinsic in the sense that it is induced by the same piezoelectric film required for acoustic excitation.

A detailed analysis of the FEM simulation shown in Fig. 1a yields interesting insights into the lateral confinement mechanisms leading to the GHz drum modes. Figure 1b shows the dependence of the field amplitude $|\textbf{u}|$ averaged over the substrate depth as a function of the frequency *f* driving the BAWR. The full spectrum consists of a sequence of lines with a periodicity in frequency $\Delta f_L =v_{L}/(2 d)$, $d=(d_\textrm{sub}+d_\textrm{film})$, equal to the splitting between the Fabry–Pérot acoustic modes confined by the top and (perfectly reflecting) bottom surface, where $v_L$ is the longitudinal acoustic velocity in the substrate and the dimensions $d_\textrm{sub}$ and $d_\textrm{film}$ are defined in the figure. The spectral section in Fig. 1b details the frequency region close to one of the Fabry–Pérot main modes. Instead of a single resonance within a free spectral range, this spectrum shows several closely spaced peaks, attributed to distinct radial acoustic modes confined in the cavity regions underneath the ZnO layer.

Panels (c) and (d) of Fig. 1 show maps of the vertical surface displacement field ($u_z$) for the resonances marked by arrows in panel (b). These maps show comparable profiles along the $\hat{z}$ directions but rather distinct radial patterns. Panels 1e and 1f display radial $u_z$ profiles for these modes averaged within the blue dashed rectangles displayed in Fig. 1c,d, respectively. The green dashed lines display the corresponding profiles recorded near the surface. Although the piezoelectric excitation is limited to the narrow, ring-shaped area underneath the BAWR, the field profiles for both modes extend over the whole region underneath the ZnO layer and decay beyond it. Note, in particular, that the profiles smoothly extend to the non-excited region at the center of the structure, thus further corroborating lateral propagation. The decay beyond the ZnO edge is attributed to the previously mentioned thickness discontinuity of the Fabry–Pérot acoustic cavity at this border, which effectively back-reflects the Lamb modes, forming a circular boundary for the drum-like vibrations underneath the ZnO film. In fact, as will be justified in “Dispersion of cylindrical Lamb-waves”, the radial mode profiles follow the expected Bessel-like radial shape $J_0( k_{r,i})$ with different radial wave vectors $k_{r,i}$, $i=0,1, 2,\dots$ [dashed lines in Fig. 1e,f, where the short oscillations arise from drum modes with a large $k_r$.].

**Fig. 2:**
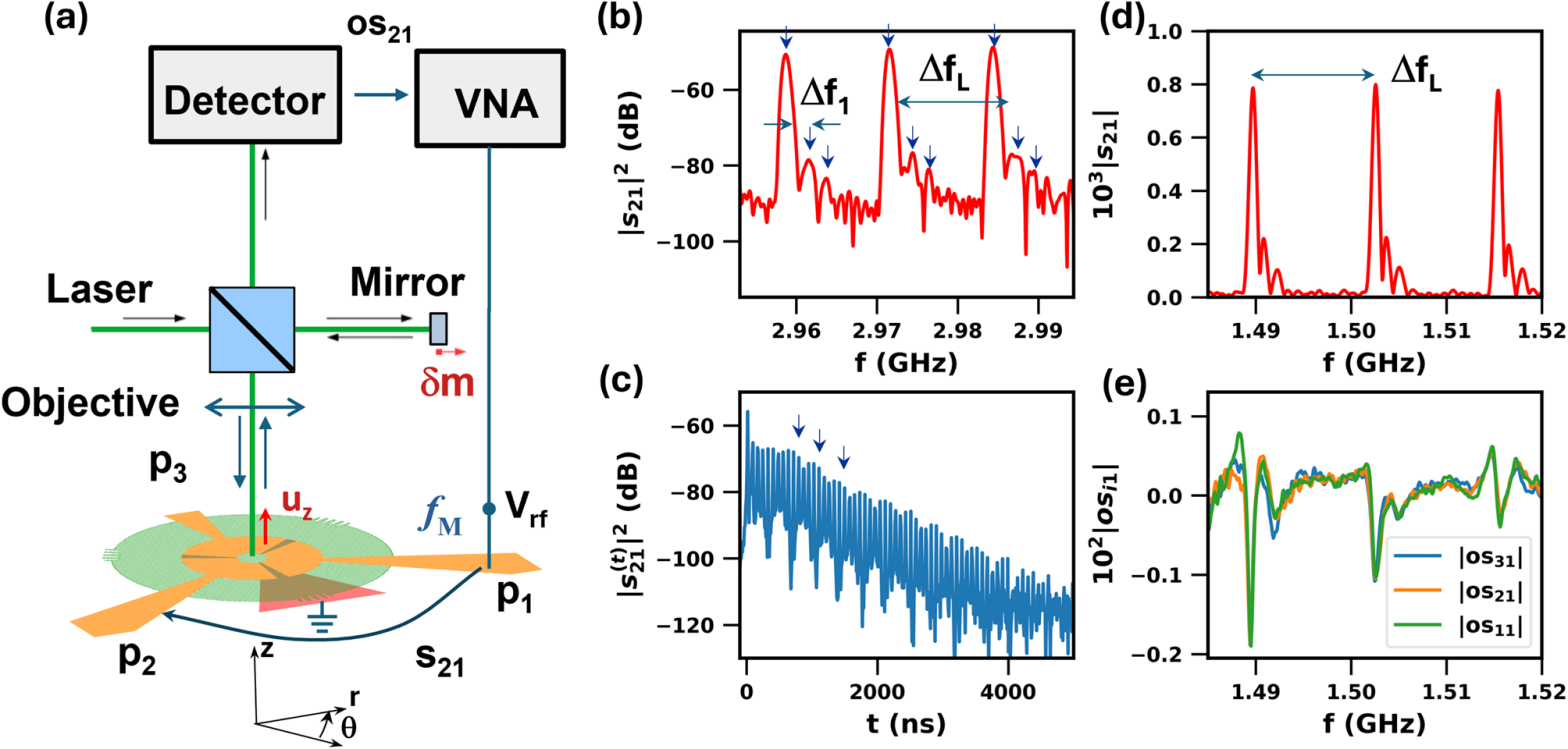
Electrical excitation of Lamb acoustic modes. (**a**) The ring-shaped BAWR has three sectors, which can be independently excited by radio-frequency fields. The electrical response is measured with a vector network analyzer (VNA). Maps of the surface displacement $u_z$ were recorded with phase resolution using a scanning Michelson interferometer with a microscope objective to focus the laser onto a $2~\mu$m-wide spot (full-width-at-half maximum) on the sample surface (see “Methods” for details). (**b**) Radio-frequency transmission ($|s_{21}|^2$ scattering parameter) between BAWR sectors $p_1$ and $p_2$ recorded in a frequency range around 3 GHz. $\Delta f_L=13.09$ MHz is the frequency difference between the modes. The arrows mark different resonances within a $\Delta f_L$ period. (**c**) Corresponding time-domain response recorded with a VNA with time-domain capabilities. The arrows show beats due to the interference of the different modes. (**d**) $|s_{21}|^2$ spectrum in the 1.48–1.52 GHz frequency range and (**e**) corresponding interferometric data recorded by exciting port $p_1$ and detecting the surface displacement $u_z$ at the center of BAWR $p_1$ (os$_{11}$), $p_2$ (os$_{21}$), and $p_3$ (os$_{31}$). The os$_{i1}$ data were corrected to remove the unstructured background. The BAWR structural dimensions are listed in the caption of Fig. 1.

### Electrical response

The experiments were performed on samples with a ring-shaped BAWR divided in three independent sectors illustrated in Fig. 2a. Figure 2b displays the rf-spectrum of the transmission parameter $|s_{21}|^2$ (corresponding to the acoustic power transmission coefficient from sector $p_1$ to sector $p_2$) over a frequency band around 3 GHz. The response consists of a series of pronounced spectral peaks separated by the free spectral range $\Delta f_L=13.09$ MHz of the Fabry–Pérot longitudinal resonances of the acoustic cavity defined by the substrate and the overlaying ZnO layer, as previously reported, e.g., in Refs. 23 and  27. The Q-factor of these electrical resonances, determined from the half-width of the $|s_{21}|^2$ peaks, is $3800 \pm 200$.

The more interesting features in the spectral response of Fig. 2b, which are also present in the simulations of Fig. 1b, is that there is not just a single resonance per $\Delta f_L$-period but rather a sequence of spectral lines (indicated by arrows in the figure). The frequency shift between the first two modes is approximately $\Delta f_1=2.53$ MHz$\ll \Delta f_L$. As will be justified in “Radially confined lamb modes”, these resonances are attributed to modes radially confined underneath the ZnO region. Similar resonances have been previously reported^23^ for modes induced by a piezoelectric thin disk on a sapphire substrate coupled to a superconducting qubit.

Figure 2c shows a time-domain representation of $s_{21}$ obtained by a Fourier transformation of the frequency domain spectrum over a frequency range of 100 MHz. This temporal data, which approximates the transmission of a short pulse excitation, consists of a series of echoes displaced by $1/\Delta f_L=76.4$ ns and an envelope modulation with a periodicity of $\sim 1/\Delta f_1 = 400$ ns (the individual echoes are not resolved in the (long) time scale of the plot). The modulated envelope is attributed to the interference of radially confined Lamb modes. The spectral features associated with confinement are observed over a wide range of frequencies extending below a GHz, as illustrated by the $|s_{21}|^2$ spectrum in Fig. 2d.

### Optical response

Different approaches have been reported for the excitation, detection, and spatial mapping of GHz acoustic modes. In many studies, longitudinal acoustic modes are excited by a short optical pulse and subsequently interrogated by a second pulse (the pump-and-probe technique^28^). This technique has been applied for the excitation and detection of Lamb modes^29,30^ as well as for the mapping of GHz modes electrically excited by a BAWR^31^. In our case, the Lamb modes were piezoelectrically excited and then detected using the Michelson interferometer sketched in Fig. 2a (see “Methods” for details). In this setup, a $\upmu$m-sized laser beam ($\lambda _{opt} = 532$ nm) is focused by a microscope objective onto a $\upmu$m-sized spot on the sample surface. The reflected beam is collected by the same objective, interferes with the reference beam reflected on the reference mirror, and is then detected by a fast optical detector (2 GHz bandwidth) connected to a vector network analyzer (VNA). A special feature of this setup is the ability to simultaneously record both the amplitude and phase of the dynamic local phase shift $\delta \phi (r,\theta )$ induced by the acoustic wave. On the highly reflective BAWR pads, $\delta \phi (r,\theta )$ is mainly determined by the displacement $u_z(r,\theta )$ of the top surface at the spot location $(r,\theta )$^20^ (i.e., the moving boundary mechanism). Under these conditions, $\delta \phi (r,\theta ) = 4 \pi u_z(r,\theta )/\lambda _{opt}$, thus providing direct access to the surface displacement amplitude (cf. “Methods”). The previous expression is also expected to be a good approximation for the surface regions exposing the ZnO film even though this film is transparent to the laser wavelength because the reflection coefficient at the ZnO/Air interface is much larger than the ZnO/sapphire one.

The relationship between the electrical and optical spectral responses is illustrated by the green curve in Fig. 2e, which displays the ratio $os_{21} \propto \delta \phi (r,\theta )/V_\textrm{rf}$. This spectrum was acquired by powering only $p_1$ and detecting mechanical vibrations—see “Methods”—at the center of sector-BAWR $p_1$. The surface displacement ($u_z \propto os_{21}$) shows dips at the principal peaks of the electrical $|s_{21}|^2$ response of Fig. 2d. As will be discussed in “Discussions”, small differences (which also depend on frequency) between the optical and electrical responses arise from the different sensitivities of the two measurement techniques to Lamb waves with different radial wave vectors.

A further remarkable feature revealed by Fig. 2e is that by powering *only* port $p_1$, one measures essentially the same interferometric response at the center of the BAWRs at ports $p_1$ (os$_{11}$, green) $p_2$ (os$_{21}$, blue) and $p_3$ (os$_{31}$, red). As will be further elaborated in the next section, this behavior is attributed to the excitation by $p_1$ of drum-like modes of cylindrical symmetry in the region underneath the ZnO film.

**Fig. 3:**
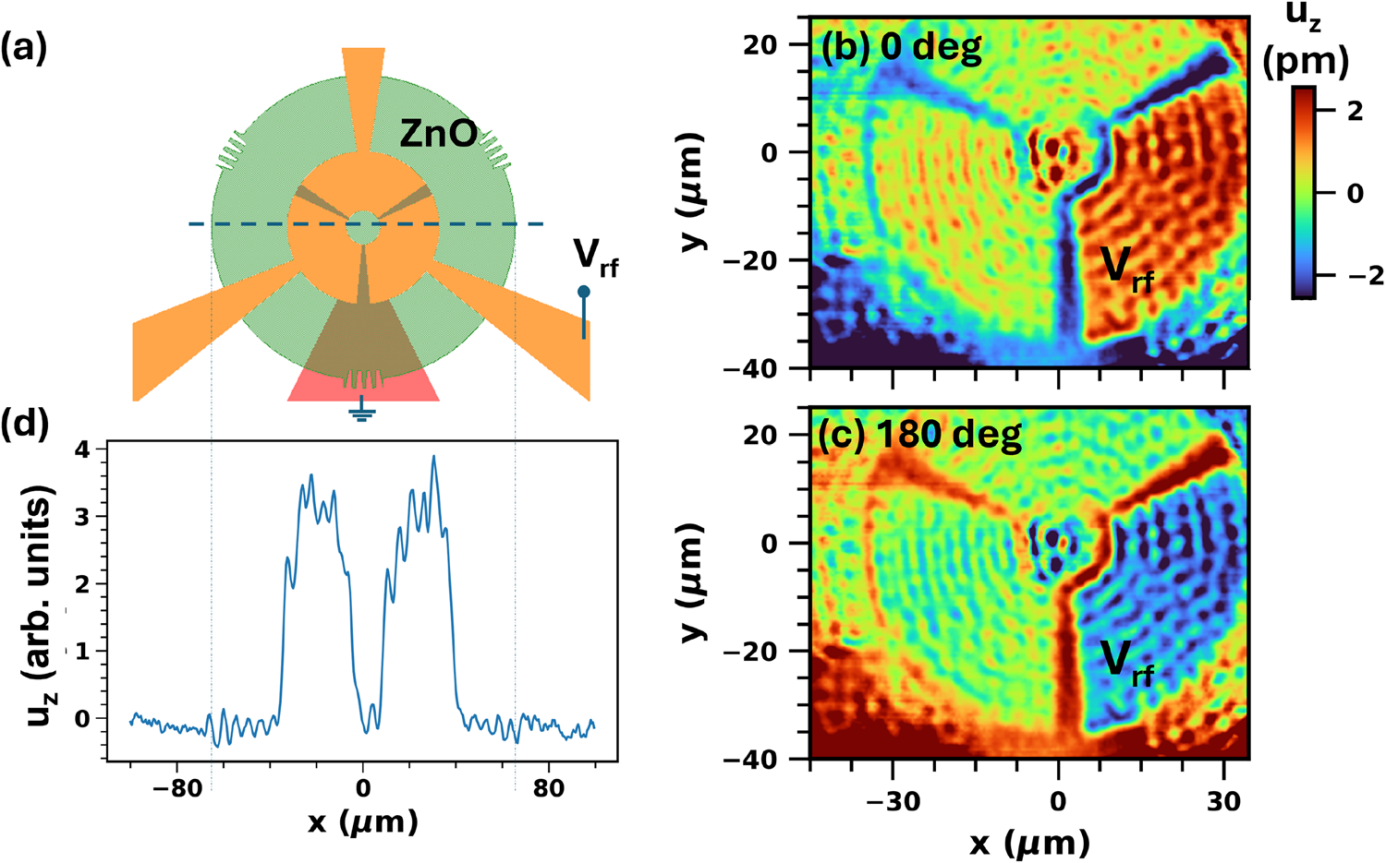
Interferometric mapping of drum modes. (**a**) Sample configuration with ring sector-shaped BAWRs with three independent sections. (**b**,**c**) Acoustic maps of the surface displacement $u_z$ obtained by exciting *only* the lower right BAWR (marked as $V_\textrm{rf}$) at the frequency of 1.5033 GHz and recording the $u_z$-component in phase and 180$^\circ$ out-of-phase with the rf excitation. The nominal rf power applied to the lower right BAWR was 20 dBm. (**d**) Line profile of $u_z$ along the dashed line in panel (**a**) showing that the short-period acoustic vibrations (i.e., the ones with ring-shaped wave fronts in **b**,**c**) are confined within the ZnO island.

### Mapping of GHz drum modes

Mappings of the acoustic field were acquired by recording $u_z(r,\theta )$ while scanning the focused beam in Fig. 2a over the sample surface. Figure 3b,c compare surface maps for the surface displacement ($u_z$(r,θ)) components in-phase and out-of-phase (i.e., with a phase shift $\phi _\textrm{rf}=180^\circ$) with respect to the rf-drive, which was applied *only* to port $p_1$. Due to the lower optical reflectivity, the signal outside the BAWR contacts is much weaker than on the metal contact of the BAWRs, but also changes sign with the detection phase. The estimated surface displacement amplitudes (color scale, cf. “Methods) are on the order of a few pm: note, however, that the acoustic field extends over the whole substrate thickness.

**Fig. 4:**
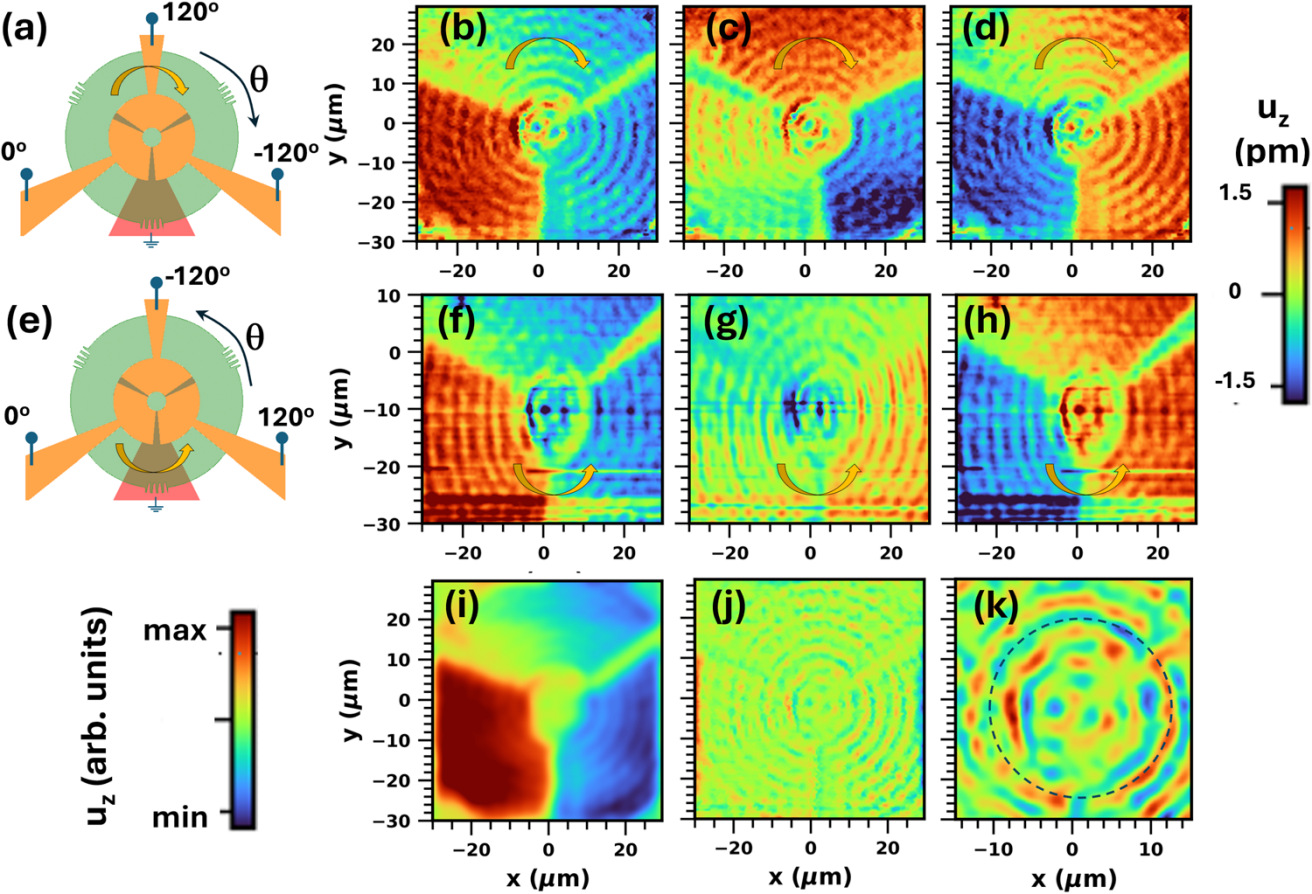
Helical drum modes. (**a**) Setup for the excitation of helical waves by driving the BAWR sectors with clock-wisely increasing phases differing by 120$^\circ$. (**b**–**d**) Profiles for the surface displacement field $u_z$ of helical modes at time instants corresponding to phases of (**b**) 0 deg, (**c**) 120 deg, and (**d**) 240 deg for $f_\textrm{RF}=1.5033$ GHz. (**e**,**h**) Corresponding results for the excitation of anti-clockwise helical waves. (**i**,**j**) Spatially filtered versions of panel (**b**) to review surface oscillations of long and short spatial periods, respectively. (**k**) Zoom view of the center region of panel (**j**) revealing the phase wrapping of the modes with short spatial periods. The color code is the same for panels (**b**–**h**) and (**i**,**j**). In panel (**k**), it was expanded by a factor of two relative to (**i**,**j**). The nominal rf power applied to each one of the BAWRs was of approx. 10 dBm.

Interestingly, the recorded interferometric pattern is almost perfectly cylindrical with essentially the same amplitude on all sectors despite the excitation of just one of the sector-shaped BAWRs. The peculiar cylindrical shape, which is corroborated by the almost identical spectral shapes in Fig. 2e, is attributed to the preferential excitation of cylindrical drum-like modes arising from the radial confinement underneath the ZnO disk. Similar to a circularly clamped drum membrane, the amplitude of the Lamb modes excited by the BAWR is enhanced when their frequency matches a drum-like eigenstate: these modes are reflected at the borders of the ZnO disk to yield a cylindrical pattern with maximum displacement amplitude at the disk center. The shape of the sector-like BAWR thus plays only a minor role in the mode lateral profile. The surface displacement should, therefore, have a Bessel-like profile $J_0(k_{r,m_r} r)$ with an effective radial wave vector $k_{r,m_r}$ given by:

1 $$\begin{aligned} k_{r,m_r} \approx m_r\frac{\pi }{r_\textrm{film}}, \quad m_r=1,2,\dots . \end{aligned}$$

where $r_\textrm{film}$ is the radius of the piezoelectric film.

**Fig. 5:**
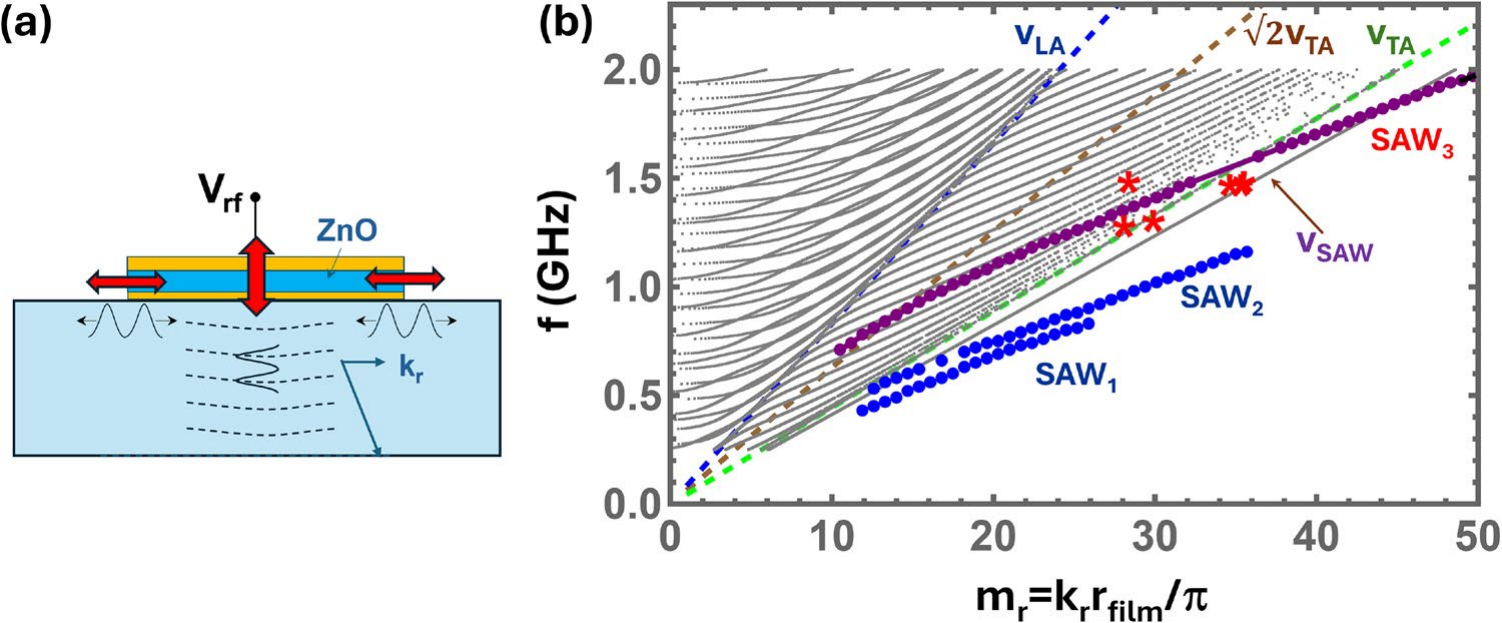
Dispersion of cylindrical Lamb waves. Dispersion of *rz*-polarized Lamb waves in a cylindrical disk of sapphire of radius $r_\textrm{film}$ with winding number $n=0$ calculated assuming an isotropic elastic model for the sapphire substrate (cf. Eq. 2). The calculations were performed for a 425 $\upmu$m-thick substrate: due to the high mode density, not all modes are shown. The in-plane wave vector $k_r$ is normalized to $\pi /r_\textrm{film}$, where $r_\textrm{film}$ is the radius of the ZnO island. The dashed lines indicate the dispersion of the pure longitudinal ($v_\textrm{L}$, Eq. SM15), transverse ($v_\textrm{T}$, Eq. SM15), Lamé ($v_\textrm{T}\sqrt{2}$, Eq. SM25), and SAW modes ($v_\textrm{SAW}$, Eq. SM17) determined for the sapphire substrate. The blue and purple dots show the dispersion of SAW modes determined for a sapphire substrate coated with a 700 nm-thick ZnO film (cf. Sect. SM2 B). The red asterisks are the corresponding experimental values for short period waves obtained from field maps (such as, e.g., those in Figs. 3 and 4).

Modes with two distinct radial periodicities, $\lambda _{r,m_r} = 2\pi / k_{r,m_r} \approx 2 r_\textrm{film}/m_r$, can be identified in the experimental maps. The first yields strong fringes with a short radial periodicity and $m_r \sim 30$, similar to the short radial wavelength structure seen in the simulations of Fig. 1e,f as well as in the spatial maps of Fig. 3. For the data in Fig. 3 recorded for $f=1.50$ GHz, this component has $\lambda _{r,m_r} = 4.3 \pm 0.2 \mu m$. This together with several other experimentally observed modes of the same type are indicted by red asterisks in the prediced Lamb-wave dispersion displayed in Fig. 5. As will be justified in “Dispersion of cylindrical Lamb-waves”, they are attributed to the excitation of Lamb modes tightly confined by the ZnO layer and its boundaries.

The maps is Fig. 3b,c only cover the region of the BAWR pads, which is smaller than the ZnO island. Interferometric maps over a wider area extending beyond the ZnO radial boundary [cf. Fig. 3a] provide the key experimental evidence for the excitation of laterally confined drum-like modes. The radial confinement of the short-period mode is clearly demonstrated in Fig. 3d, which displays an interferometric line scan extending over the whole diameter of the ZnO disk [cf. dashed line in Fig. 3a]. The larger oscillations are again due to the modes localized near the surface. $u_z$-oscillations on the BAWR contact areas also show the higher reflectivity of these metal-coated areas compared to that of the ZnO-coated substrate. The $u_z$-oscillations propagate over several tens of $\upmu$m until they reach (and get reflected from) the border of the ZnO disk and decay away from it.

A second mode type with a much longer surface wavelength $\lambda _{r,1} \sim 2 r_\textrm{film}$ appears in Fig. 3b,c as a faint background superimposed on the fringes of the short-period mode [the latter is most clearly seen by spatially filtering the signal, cf. Fig. 4i]. The interference of these two (short- and long-period) modes builds the strong displacement antinode at the center of the pattern. Due to the smaller surface displacement amplitudes, the interferometric line profile of Fig. 3d cannot clearly detect the radial confinement of the long-period mode.

### Helical drum modes

The sector-like BAWR configuration can generate GHz helical waves when all sectors are powered with rf-voltages with phases $\phi _\textrm{rf}$ that increase by 120 deg in the clock-wise direction [cf. Fig. 4a]. Panels 4b–d show interferometric maps of the optical phase (or surface displacement) recorded for this configuration at time instants corresponding to these phases. As discussed in Ref. 20, the mapping procedure is analogous to the holographic determination of the phase cross-section of an extended optical beam with $\upmu$m spatial and sub-ns time resolutions. If the acoustic vortex region would be illuminated by an extended optical beam (i.e., with dimensions compared to the one of the acoustic vortex), the phase cross-section of the reflected beam would then acquire the same phase pattern $\delta \phi (r,\theta )$. The maps thus directly show that the moving surface transfers angular momentum to the reflected optical beam.

Panels 4f–h display the corresponding plots when the BAWRs are driven in a counter-clockwise direction [cf. Fig. 4e]. The wave rotation can be readily identified by following the changes in the time-evolved interferometric maps around a clockwise or counter-clockwise path [e.g., as indicated by the curved arrows in Figs. 4b,f]. The observed phase wrapping results in the creation of vortices at the center of the pattern with topological charges. In particular, the acoustic vortices have topological charges $\ell =1$ and $\ell =-1$ for anti-clockwise and clockwise rotations, respectively. Videos showing the complete time evolution of the clockwise and counter-clockwise rotating maps are included in Section SM4.

The helical displacement profiles of Fig. 4b–d,f–h are composed of vibrations with both long and short radial wave vectors. In order to distinguish these two contributions, Fig. 4i,j display the same data as in panel 4b after spatial filtering wave components with long and short radial periods, respectively. The long period mode shown in Fig. 4i has the strongest amplitude variation with the azymuthal angle $\theta$, forming a vortex at the center of the pattern.

The short-period oscillations [cf. Fig. 4j] have, in contrast, a weaker displacement amplitude. Their helical character becomes evident when plotted over the smaller region shown in Fig. 4k. By tracing a circular path (indicated by the dashed circle) around the vortex, one can easily confirm that the phase of the wavefronts shift by 120$^\circ$ between neighboring BAWRs with helical polarity dictated by the rotation direction for phase increase. The complex shape of the vortex at the center arises from the discrete phase evolution with $\theta$. A better approximation for a spiral wave front shape can be obtained by increasing the number of BAWR sectors.

## Discussions

We have demonstrated the piezoelectric generation of drum-like Lamb waves propagating over several tens of $\upmu$m, which can be radially confined using overlayers on the substrate. Furthermore, we have shown that GHz Lamb modes with positive and negative OAM can be excited by an array of BAWRs driven by synchronized rf phases. The modes with acoustic angular momentum can be mapped with optical techniques, thus demonstrating the transfer of angular momentum to a light beam.

Despite the monochromatic excitation, the BAWRs employed in the present studies generate Lamb waves with different radial wavevectors. Phenomenologically, the excitation of different radial wavevectors can be understood with the help of Fig. 5a. The rf-voltage applied to the BAWR electrodes induces an oscillating stress field in the piezoelectric film, which preferentially launches longitudinal modes towards the substrate. If the lateral size of the BAWR is small, it will, together with wave diffraction, impart a small radial wave vector component $k_{r,m_r}, m_r\sim 1$ to the launched wave with amplitude dictated by the BAWR size (cf. Eq. 1). These waves then bounce back and forth at the top and bottom sample boundaries to form propagating Lamb modes with a small $k_{r,m_r}$.

The deformation of the piezoelectric film along *z* is, in addition, accompanied by a radial deformation and the creation of shear stresses at the lateral boundaries of the BAWR. These deformations can launch Lamb modes with a radial wave vector satisfying Eq. (1) for large $m_r$, corresponding to wavelengths considerably smaller that the BAWR lateral dimensions. As will be discussed below, Lamb waves with large $k_r$ can become fully confined to the substrate surfaces, eventually resulting in the formation of surface acoustic waves (SAWs). Finally, if the BAWRs are driven at different phases as in Fig. 4, these phases will be imparted to the waves, leading to the formation of helical modes.

The electrical (i.e., s-parameters) and optical (interferometry) field mapping techniques are expected to be most sensitive to SAW-like Lamb modes with strong field localization near the surfaces. While interferometry can resolve these short wavelength modes, the electrical detection integrates the contributions of waves with radial wavelengths less than approximately twice the BAWR dimensions, thus making it primarily sensitive to Lamb modes with large radial wavelengths. These differences in detection sensitivity can qualitatively account for the different profile shapes in Fig. 2d,e. In the case of waves with non-zero angular momentum, the spatial filtering technique employed in Fig. 4i,j shows that interferometry can, in this case, detect the helical character of waves with both small and long spatial radial periods.

In order to quantitatively analyze the experimental results, we first calculate in the next section the dispersion of cylindrical Lamb waves propagating in a disk. We then address the impact of the ZnO overlayer on these waves (“ZnO-induced surface modes”) as well as on their radial confinement (“Radially confined lamb modes”).

### Dispersion of cylindrical Lamb-waves

Lamb-waves in plates have been the subject of several studies (see, e.g., Refs. 32 and 33), mostly addressing 1D Lamb modes propagating along a single direction. Only a few studies have considered the propagation of Lamb waves in the cylindrical geometry relevant to the present studies of Fig. 1a^34^. Here, we summarize the main analytical results for modes propagating in an isotropic disk limited by free surfaces at $z=\pm d/2$, which will be assumed to model the sapphire substrate (the sapphire acoustic anisotropy thus will be ignored, cf. Sect. SM1). Details of the model can be found in the supplementary Sect. SM2.

The relevant Lamb modes for our experimental configuration are those that can be piezoelectrically excited and, thus, associated with a non-vanishing surface displacement profile. Lamb modes with angular frequency $\omega$ and radial wave vector $k_r$, $\textbf{u}(r, \theta , z)$ satisfying this constraint can be expressed as a superposition of a longitudinal (L) and a transverse (T) wave both polarized in the $\hat{r}\hat{z}$ plane and with the same wave vector component $k_r$ along the radial direction. The *L* and *T* polarization components are relative to the propagation direction defined by the wave vector $(k_r, k_i), (i=L,T)$ defined by Eq. SM15. The dispersion relation of these modes is given by the solutions of the following equation:

2 $$\begin{aligned} \left[ \frac{\tan \left( \frac{d k_T}{2}\right) }{\tan \left( \frac{d k_L}{2}\right) } \right] ^{i_s} + \frac{4 k_L k_T k_r^2}{ \left( k_T^2 - k_r^2\right) \left[ (v_L/v_T)^2 (k_L^2+k_r^2 ) - 2 k_r^2\right] } = 0, \end{aligned}$$

where $v_L$ and $v_T$ are, respectively, the longitudinal and tranverse velocities and $k_p^2 = \left( {\omega }/{v_p}\right) ^2 - k_r^2$ ($p=L,T$) the wave vectors of their longitudinal and transverse components along $\hat{z}$. The exponent $i_s=\pm 1$ is related to the reflection symmetry of the mode envelopes relative to center plane $z=0$ of the disk: modes with $i_s= + 1$ are symmetric while those with $i_s= - 1$ are anti-symmetric (see Sect. SM2 A 2 for details). The displacement field for the solutions with $i_s= 1$ is given by:

3 $$\begin{aligned} \textbf{u}^n_{k_r}(r, \theta , z)= & e^{i n \theta } B_e \left( \begin{array}{c} r^{-1} \left[ k_r r J_{n-1}(k_r r)-n J_n(k_r r)\right] \left[ (k_T^2-k_r^2)\cos (k_L z) - 2 k_L k_T\cos ( k_T z ) \right] \\ - k_L J_n(k_r r) \left[ \sin (k_L z) + 2 \frac{k_r^2}{(k_T^2-k^2_r)}\sin (k_T z) \right] \end{array} \right) \left( \begin{array}{c} {\hat{r}}\\ {\hat{z}}\\ \end{array} \right) , \end{aligned}$$

where $B_e$ is the mode amplitude. The same expression applies for the $i_s=-1$ modes by interchanging $\sin \rightarrow \cos$ and $\cos \rightarrow \sin$. It is interesting to note that while the dispersion of these modes does not depend on the winding index *n*, their spatial profiles (or wave functions) do.

The black lines in Fig. 5b show the Lamb-wave dispersion for the elastically isotropic approximation for sapphire determined using Eq. 2. $k_r$ is normalized to $k_{r,1}=\pi /r_\textrm{film}$, where $r_\textrm{film}=70~\upmu$m is the radius of the piezoelectric film (this normalization will facilitate comparison with the Lamb modes of a disk with finite radius $r_\textrm{film}$ in “Radially confined lamb modes”). Similarly to 1D Lamb modes (i.e., propagating along a single direction, cf. Ref. 33), the dispersion for small $k_r$’s (i.e., $k_r\ll k_L, k_T$) can be approximated by the superposition of the quadratic dispersions of the transverse and longitudinal wave components given by:

4 $$\begin{aligned} \omega _{k_r} = v_p \sqrt{k^2_p + k_r^2} \approx m_p v_p \frac{\pi }{d}\left[ 1+\frac{1}{2} \left( \frac{k_r}{k_p}\right) ^2\right] , \end{aligned}$$

where $$p=L,T$$ and $$m_p=1,2,\dots$$.

The shape of the modes depend on the relationship between $k_r$ and the frequency *f*. For $k_r$ in the range $[0,2\pi f/v_L]$, $k_L$ and $k_T$ are both real and the transverse and longitudinal mode components extend over the whole substrate. The dispersion branches can, however, interact and anti-cross, leading to a complex dispersion shape. Furthermore, the solutions of Eq. (4) fullfil the condition $k_p d/2=m_i$, for both $p=L,T$, i.e., both the transverse and longitudinal wave components have an integer number of half cycles within the substrate. For the present experimental situation, $m_p>30$ is a large number. Under these conditions, it can be easily shown that the frequency spacing between the Lamb modes around a given frequency becomes $\Delta f_\textrm{Lamb} \approx m_\textrm{GCD} v_T/(2 d)$, where $m_\textrm{GCD}$ is the greatest common divisor (see, e.g., https://en.wikipedia.org/wiki/Greates_ common_divisor) (or factor) of $m_L$ and $m_T$.

For increasing $k_r$, the mode frequencies asymptoptically approach first the linear dispersion of bulk longitudinal and then of the bulk transverse modes propagating along the radial direction, which are reproduced by the blue and green dashed lines in Fig. 5, respectively, with phase velocities $v_L$ and $v_T$. For frequencies, *f*, satisfying $2\pi f/v_L<k_r<2\pi f/v_T$, the wave vector component $k_T$ remains real, but $k_L$ becomes imaginary. Such transverse modes extend over the whole substrate, while the longitudinal modes decay away from the surfaces. The brown dashed line shows the dispersion of the so-called Lamé modes^33^, which are transverse vibrations polarized along $\hat{z}$. Finally, for $k_r>2\pi f/v_T$ all branches converge to the single SAW branch with an acoustic displacement fully confined to the surface near region. The dispersion equation for cylindrical SAWs is given by Eq. SM17.

**Fig. 6:**
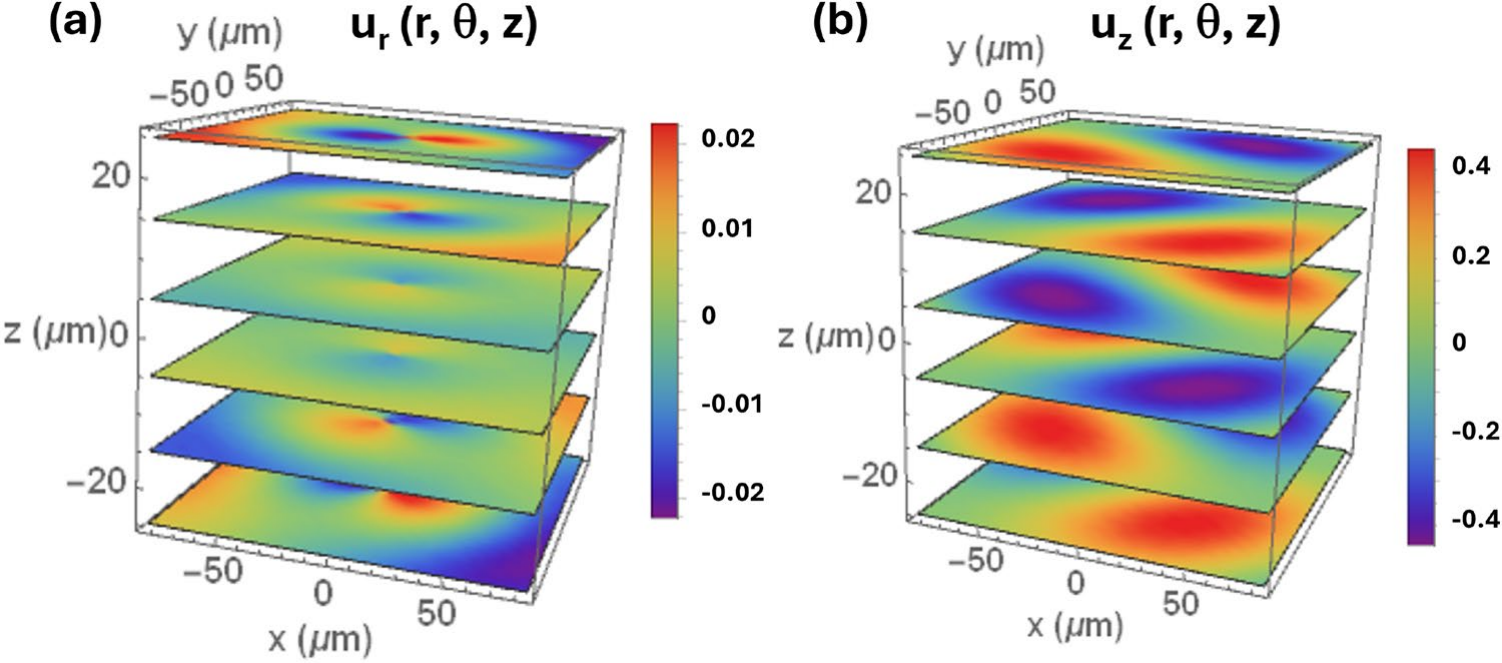
Long-wavelength helical Lamb wave profiles. Displacement field along (**a**) $\hat{r}$ and (**b**) $\hat{z}$ calculated using Eq. (3) for a quasi-longitudinal mode with radial wave vector $k_r=2\pi /r_\textrm{film}$ and winding number $n=1$.

The solutions of Eq. (4) for modes with longitudinal character with a small radial wave vector and, thus, polarization close to $\hat{z}$ are of particular interest for us. These modes can be efficiently excited and detected by the BAWR since their strain field near the surface is almost colinear with the applied rf field, a configuration which yields the highest electromechanical coupling (cf. Sect. SM2 A 2). Figure 6a,b display maps for the radial and vertical components of the displacement field for such a Lamb wave with a winding number $n=1$ and a small wave vector component (corresponding to $k_r=k_{r,1}=\pi /r_\textrm{film}$). Due to the small $k_r$, the displacement amplitudes along $\hat{z}$ are much larger than the ones in the radial direction. The displacement field rotates counterclockwise both in time and as a function of depth creating a vortex at the center of the pattern.

The rotation pattern at the sample surface of Fig. 6 approximates the interferometric maps for different phases in Fig. 4. Here, it is important to recall that the angular phase profile created by the three sector-BAWRs is not continuous but rather consists of three discrete steps [cf. 4k]. In order to reproduce the experimental configuration, a superposition of modes $\textbf{u}^n_{k_r}(r, \theta , z)$ with different winding numbers *n* must be applied.

### ZnO-induced surface modes

The ZnO overlayer provides the necessary piezoelectric response to electrical excitation and the thickness discontinuity for the radial confinement of the Lamb modes. The analysis of cylindrical Lamb modes in the previous section disregards this layer, which is very thin compared to the substrate. This approximation is valid for Lamb modes with small $k_r$, which extend over the whole substrate. For large wave vectors $k_r\ge k_T$, in contrast, both $k_L$ and $k_T$ become imaginary (cf. Eq. 2) and the Lamb modes turn into SAWs with fields fully confined near the surface: the ZnO overlayer can, in this case, significantly affect the acoustic propagation properties. In particular, since the acoustic velocities of ZnO are significantly lower than the sapphire ones, the ZnO layer effectively creates a surface waveguide for acoustic waves that can support SAW-like Lamb modes with different transverse profiles.

To address the effects of the ZnO layer, we numerically calculated the dispersion of SAW-like Lamb modes of a ZnO-coated sapphire substrate (see Sect. SM2 B for detail). The results are shown by the blue and purple dots in Fig. 5b. The calculations were performed for modes propagating along one dimension (i.e., the cylindrical character was not considered) and yield not only the frequency but also the piezoelectric generation efficiency (i.e., the electromechanical coupling efficienty $k^2_\textrm{SAW}$ of the modes. Figure 5b only includes modes with a moderate to high electromechanical coupling. SAW$_1$ and SAW$_2$ are modes with a moderate electromechanical coupling. SAW$_3$ is a true SAW mode for large $k_r$. However, this mode becomes a pseudo-SAW with a large electromechanical coupling ($k^2_\textrm{SAW}$ up to 6%) when it enters the dispersion range of extended Lamb modes of the sapphire substrate for small $k_r$’s. The short-period modes detected in Figs. 3 and 4 are attributed to this mode: indeed, the measured oscillation periods (red asterisks in Fig. 5) agree very well with the mode dispersion.

### Radially confined Lamb modes

**Fig. 7:**
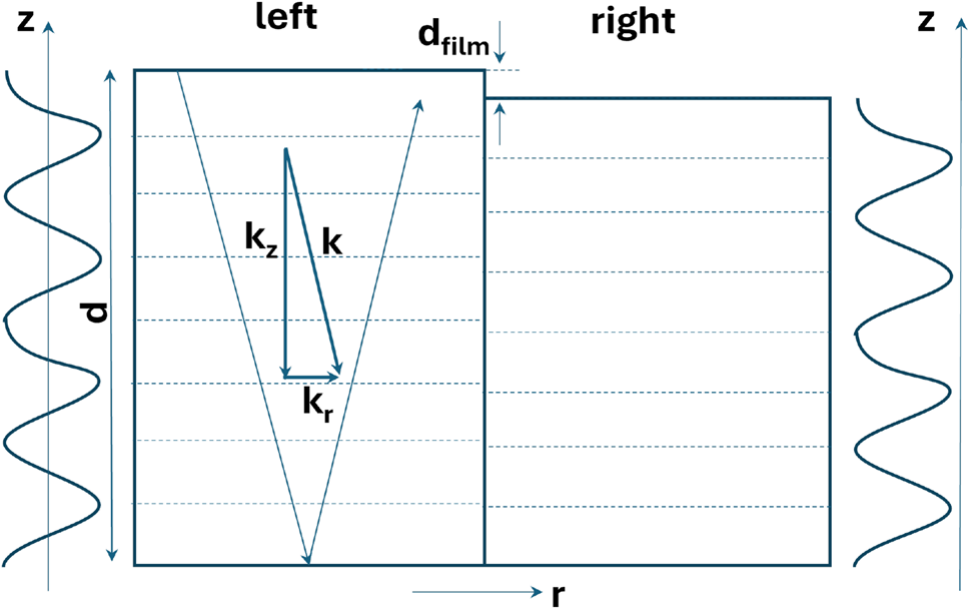
Radial confinement mechanism. Propagation of Lamb waves with as small in-plane wave vector component $k_r$ across the interface between two plates with different thickness $d_\textrm{film}\ll d$. The side plots displays the wave profile at the left and right sides for frequencies $f_m$ and $f_m+\Delta f_m$, respectively, satisfying the boundary conditions at the top and back surfaces. The corresponding displacement anti-nodes are indicated by the dotted horizontal lines.

We now address the radial confinement of the piezoelectrically excited modes, which promotes the formation of drum-like Lamb modes underneath the ZnO film. The thickness discontinuity, $d_\textrm{film}$, introduced by the ZnO layer creates Fabry–Pérot acoustic cavities with different thicknesses $d=d_\textrm{sub} + d_\textrm{film}$ and $d_\textrm{sub}$ (cf. Fig. 7) and hinders mode propagation between these two regions. To estimate the acoustic reflection coefficient at the lateral interface, we assume that an essentially longitudinal Lamb eigenmode is excited on the left Fabry–Pérot cavity with frequency $f_m$ and wave vector $\textbf{k} = (k_r, k_z)$ with a small radial component $k_r$. The in-plane velocity for that mode can be approximated by:

5 $$\begin{aligned} v_r \approx v_{L} k_r/|k_z|. \end{aligned}$$

The side panels of Fig. 7 display profiles for the wave displacement $u_z(r, z)$ eigenmodes of the left and right cavities with the smallest frequency difference $\Delta f_m$. These eigenmodes must satisfy the boundary conditions at the top and back surfaces for frequencies $f_m$ and $f_m+\Delta f_m$, respectively. These boundary conditions require an integer number ($m_L$) of acoustic half cycles in each cavity, i.e., the mode frequencies are such that both the top and bottom surfaces are displacement antinodes (dotted horizontal lines in Fig. 7).

The most interesting case occurs when the overlayer thickness $d_\textrm{film}$ is much smaller than half of the wavelength along *z*, i.e., $|d_\textrm{film}|<d/(2 m_L) = v_{L}/(4f_m)$. The eigenmodes with closest frequencies in the two cavities will then have wave vectors with z-components given by $(m_L-1) \pi /d$ and $m_L \pi /(d - d_\textrm{film})$. By using Eq.  (5), it is then straightforward to show that the relative mismatch in frequency ($\Delta f_m/f_m$) or radial velocity ($\Delta v_r/v_r$) along the in-plane direction becomes:

6 $$\begin{aligned} r_f = \frac{\Delta f_m}{f_m} =\frac{\Delta v_r}{v_r} = -\frac{d_\textrm{film}}{2 d}. \end{aligned}$$

This approximation applies for $d_\textrm{film}< \frac{d}{m_L} = \frac{v_{L}}{4f_m}$. For a given $d_\textrm{film}$, the maximum frequency mismatch (and reflection coefficient) is then inversely proportional to the frequency, and, for a fixed frequency, reduces with increasing substrate thickness.

For our samples, $r_f = \Delta f_m/f_m \approx 0.08\% = 1/1250$. This value exceeds the measured inverse quality factor $1/Q=1/3800$ of the extended Lamb modes by approximately a factor of 3. The high Q acoustic cavities reduce the overlap between the frequency spectra at the two sides of the lateral interface, leading to an efficient acoustic reflection at the ZnO boundaries [cf. Fig. 1c,e]. High Q’s are thus essential for an efficient confinement yielding drum-like Lamb modes. Finally, while derived for extended Lamb waves with a small wave vector ratio $k_r / k_L$, Eq. (6) also applies for SAWs provided that one replaces the thickness *d* by the penetration depth $\approx \lambda _\textrm{SAW}$ of the acoustic field, where $\lambda _\textrm{SAW}$ is the SAW wavelength. This yields significantly larger reflections for SAWs compared to extended modes.

In the absence of radial confinement, there is no restriction on the radial wave vector $k_r$ and the Lamb modes have a continuous frequency spectrum [cf. Fig. 5b]. The situation changes if the modes become confined underneath the ZnO film: the available values for $k_r$ will then depend on the radial boundary conditions at the boundaries $r=r_\textrm{film}$ of the confinement area. A closed analytical solution for the confined field can, in general, not be derived. A very good approximation for small $k_r$ (i.e., $k_r\ll k_T, k_L$) is obtained by imposing at $r=r_\textrm{film}$ the constraint:

7 $$\begin{aligned} J_n(k_r r_\textrm{film})=0\rightarrow & \nonumber \\ k_{r,m_r}= & \frac{1}{r_\textrm{film}}Z_{b_n}(m_r) \approx \sqrt{m_r^2 + n^2} \frac{\pi }{r_\textrm{film}}, \end{aligned}$$

where $m_r = k_r r_\textrm{film}/\pi$ and $Z_{b_n}(m_r)$ is the zero of $J_n$ of order $m_r$. The approximation on the right hand side is justified in Sect. SM2 C. For $n=0$, one retrieves Eq. (1): the confined solutions are then the dispersion points in Fig. 5 for which $m_r$ is an integer, i.e., $m_r=1,2,\dots$. Interestingly, this approximation applies for both a stress-free as well as for a clamped lateral surface at $r=r_\textrm{film}$.

The red dashed lines superimposed on the simulation results of Fig. 1e,f display profiles for the $u_z(r) \propto J_0(k_{r, m_r})$ (cf. Eq. 3) calculated using $k_{r,m_r}$ values determined using Eq. (7) for $m_r=1$ and $m_r=2$, respectively. The Bessel function approximation reproduces well the finite element field results.

We now proceed to the determination of the frequency shifts induced by the radial confinement. By combining Eqs. (4) and (7), we obtain the following expression for the frequency of the drum modes of small $k_r$ with longitudinal and radial indices $m_L$ and $m_r$, respectively:

8 $$\begin{aligned} f(m_L, m_r) = f_{m_L} + \frac{v^2_L Z_{b_n}^2(m_r) }{8 f_{m_L} \pi ^2 r_\textrm{film}^2},\,\text{where}\, f_{m_L} = m_L v_L \frac{\pi }{d}. \end{aligned}$$

The radial drum modes thus add a series of resonances to the longitudinal substrate modes (index $m_L$) with frequency spacing dictated by the radius of the ZnO film.

We attribute the extra resonances observed in the electrical response [cf. Figs. 1b and 2b] close to main longitudinal modes to the excitation of the radial drum modes given by Eq. (8). In fact, the splitting $\Delta f_1=7$ MHz between the first two modes in Fig. 1b is very close to the splitting of 7.7 MHz calculated from Eq. (8) for $f_{m_L} = 1.04$ GHz. Furthermore, the splitting $\Delta f_1=2.5$ MHz in Fig. 2b agrees very well with the predicted value of 2.7 MHz for $f_m=3$ GHz.

The prediction for the splitting between the third and the first lines of 7.5 MHz, however, exceeds the measured value of 4.4 MHz. The discrepancy may be associated with an increased interaction between the dispersion branches in Fig. 5, which is not accounted by the simple model of Eq. (8). Further work will be required to explain these deviations.

The observation of the resonances in the electrical reflection together with the quantitative agreement with the model thus provide a further unambiguously evidence for radial confinement of the small $k_r$ modes. Here, we remind ourselves that, in contrast to the radial confinement of short period vibrations established by Fig. 3d, the radial confinement of long-period vibration could not be established by interferometric measurement due to their small surface displacements.

## Conclusions

We have demonstrated a novel concept for the piezoelectric generation of drum-like helical acoustic Lamb waves in the GHz range. The concept exploits the vertical confinement via acoustic reflections at the back side of the substrate as well as the radial confinement by substrate thickness variations. We have shown here that the concept can be easily extended to the generation of helical Lamb modes, which, via acousto-optical interactions, should in future be able to produce light beams with tunable OAM.

The Lamb-wave approach enables reaching considerably higher frequencies than those so far reported for helical beam generation using arrays of interdigital SAW transducers (IDTs)^19^ or IDTs with ring^36^ or spiral-like shapes^17^. On the one hand, these SAW-based processes can also profit from the lateral confinement using overlayers introduced here. On the other hand, the Lamb-wave approach can be straight-forwardly extended to the spiral-shaped BAWR geometries.

While for the spiral-type geometries the helicity sign is fixed by the structure shape, approaches using arrays of SAW’s or BAWR’s enable dynamic control both the magnitude and polarity of the helicity, thus offering extra flexibitity for signal processing. Due to the finite number of transducers, these approaches, however, normally generate a superposition of modes with different winding numbers *n*. One of the challenges is then to increase the number of phase-synchronized transducers for the generation of modes with a well-defined *n*.

The Lamb-generation and radial confinement rely on the formation of a high-Q acoustic cavity in the substrate: they thus require substrates with low acoustic absorption. The radial confinement can be substantially enhanced by increasing the thickness contrast ratio between the confinement region and the surroundings (cf. Eq. 6), e.g., by using thicker overlayers or reducing the substrate thickness. Alternatively, the acoustic field can be vertically confined by using underetching or via the use of distributed Bragg reflectors (DBRs), which have become conventional processes for the BAWR technology.

We now briefly address prospects for the enhancement of the acousto-optical generation efficiency of a helical optical beam. Due to the small displacement amplitudes ($\delta \phi (r,\theta )\ll \lambda _\textrm{opt}$), the phase modulation $\delta \phi (r,\theta )$ imprinted via reflection on the acoustic vortices is very small (i.e., $\ll 2\pi$) and, thus, the ammount of transferred OAM is not sufficient to form optical vortices^20^. We note, however, that optical helicity modulations much smaller than $2\pi$ are already sufficient for several applications. As an example, Ref. 20 shows that a quasi-orthogonal helical basis for information encoding can already be obtained from helical acoustic waves with surface displacements $u_z$ of a tenth of the optical wavelength. The acoustic fields can be considerably enhanced by optimizing the transducers. Furthermore, appropriate designs can exploit the acousto-optical coupling based on dynamic diffraction within the substrate material. Similar to work in optical fibers, one can engineer specific resonances between optical and acoustic wavelengths in order to create a dynamical Bragg pattern controlled by the strain wave via the optoelastic effect^18^. Large modulation amplitudes can, for instance, be achieved by Bragg back-scattering of an incoming light beam in the standing acoustic mode formed in the substrate. Phase matching for the acousto-optical interaction requires, in this case, an optical wavelength $\lambda _\textrm{opt} = 2 n_{ref} v_{LA} /(mf_\textrm{BAW}$), where $n_{ref}$ is the substrate refractive index and $m=1, 3, \dots$ the diffraction order. For the optical wavelength $\lambda _\textrm{opt}=532$ nm employed in the present experiments, this condition can only be fulfilled for very high acoustic frequencies (i.e., approx 74 GHz and 25 GHz for first and third-order diffractions, respectively). For an optical wavelength in the near-infrared $\lambda _\textrm{opt}=1550$ nm, however, the third-order Bragg condition is satisfied at a much lower frequency $f_\textrm{BAW}=8.5$ GHz.

The ability to laterally confine Lamb modes can be exploited for the generation of two-dimensional (2D) resonators or phononic crystals on a solid substrate, like the ones realized on membrane or beam structures^37^ as well as to investigate concepts for enhanced optomechanical coupling in these structures^38–40^. Finally, a particularly interesting approach exploits the simultaneous confinement of light and vibrations formed in semiconductor microcavities^41–44^. The strong overlap between the optical and vibrational fields ensures a large acousto-optical coupling, which can be further enhanced by introducing quantum-well excitonic resonances to form light emitters of $\upmu$m sized based on exciton polaritons and exciton polariton condensates^45^. Modulation of these light emitters by piezoelectrically stimulated bulk acoustic vibrations has been demonstrated^45^: the helical Lamb-wave concepts introduced here can impart OAM to the emission from confined polaritons and their Bose–Einstein-like condensates. The latter provide a powerful approach for the efficient generation of chiral optical beams with tunable OAM. In addition, it enables exploitation of the rich physics of rotating polariton condensates under an acoustically induced stirring, which has so far only been addressed using optical stirring methods^46–48^.

## Methods

### Sample structure and fabrication

The devices [cf. Fig. 2a] were fabricated starting from the bottom BAWR metal contact, a 10 nm Ti/30 nm Au layer stack deposited on a 425 $\upmu$m-thick c-plane sapphire substrate. The bottom BAWR contact was then coated with a $d_\textrm{film}= 700$ nm-thick layer of textured piezoelectric ZnO film capped with a 20-nm-thick protective SiO$_2$ layer using rf-magnetron sputtering at 150$^\circ$. This ZnO thickness yields a center resonance frequency for longitudinal acoustic (LA) modes of approx $v_{L}/(2 d_\textrm{film})\sim 4$ GHz. The BAWRs are, however, able to excite vibrations over a wide range of frequencies extending down to less than 1 GHz. The ZnO layer was then subsequently etched to form a circular disk with radius $r_\textrm{film}$.

In the final step, the top contact, a 10 nm Ti/30 nm Al/10 nm Ti layer stack was fabricated using a photolithographic lift-off process. BAWRs with different dimensions were fabricated and tested: we will report here only on a device with the geometric parameters given in the caption of Fig. 1.

### Numerical simulations

Finite-element simulations of the sample structure were performed using COMSOL Multiphysics. The ZnO film and the sapphire substrate were defined as isotropic, i.e., ignoring the in-plane acoustic anisotropy of the trigonal sapphire substrate (cf. Sect. SM1). Under this assumption, the acoustic displacement field $\textbf{u}$ can be conveniently determined in cylindrical coordinates using a two-dimensional model.

### Acousto-electrical and -optical responses

The rf response of the BAWRs was investigated using a vector network analyzer (VNA) to measure the electrical scattering parameters $s_{ij}$ [cf. Fig. 2]. The sector-like BAWRs [cf. Fig. 2a] are excited by independent radio-frequency (rf) drives. This configuration enables probing each BAWR individually, which is advantageous for the determination of the acoustic coupling between them, as well as for the excitation of helical waves. For the latter, the BAWRs were driven with rf phases differing by 120 degrees between neighboring sectors, opportunely controlled using an rf phase shifter (cf. Sect. SM3).

Maps of the vertical surface displacement $\delta z$ were recorded using a scanning Michelson interferometer based on a $\lambda _{opt}=532$ nm single-mode laser with a spatial resolution of approximately $2~\upmu$m, as determined by the full-width-at-half-maximum of the spot focused onto the sample surface [cf. setup of Fig. 2a]. In order to obtain information about the amplitude and phase of the displacement, the interferometric signal at the BAW frequency ($V^{int}_\textrm{BAW}$) is detected by a fast photodetector (bandwidth of 2 GHz) connected to the VNA delivering the rf drive. The stabilization of the interferometer is achieved by vibrating the mirror in the reference arm at 15 kHz with a fixed amplitude of $u_M=20$ nm using a piezoelectric actuator. The interferometric signal due to the mirror oscillation ($V^{int}_\textrm{M}$) is then demodulated using a lock-in amplifier to provide the feedback signal for the piezoelectric actuator. The same signal also enables the estimation of the absolute phase shift induced by the acoustic surface displacement. If this phase is determined solely by the normal surface displacement $u_z$, then $u_z = u_M V^{int}_\textrm{BAW}/V^{int}_\textrm{M}$. The absolute values for $u_z$ quoted in the main text are estimations determined using the nominal gains of the electronic components rather than really calibrated values.

The sample is mounted on a piezoelectric xy-table. For the field maps, the sample was scanned at steps of approx. $0.25~\upmu$m. The interferometric signal was recorded at each point with a typical acquisition time of 1 s per point, limited by the time-constant of the stabilization feedback loop. The total acquisition time for a $40\times 40~\upmu$m$^2$ is of a few hours.

## Supplementary Information

Supplementary Information.

## Data Availability

The measurement and numerical simulation data that support the findings within this study are included either within the main text or the Supplementary Information, and are available upon request.
