# Supplementary material for: Method for GHz optical helicity modulation by acoustic drum modes on a chip
Source: Sci Rep. 2025 Nov 7;15:38991. doi: 10.1038/s41598-025-26587-9 (PMC12594992; doi:10.1038/s41598-025-26587-9)
Supplement: Supplementary file 1 — Supplementary Information. [file 41598_2025_26587_MOESM1_ESM.pdf]

SUPPLEMENTARY INFORMATION:
Method for GHz optical helicity modulation by acoustic drum modes on a chip

N. Ashurbekov,[1] I. dePedro-Embid,[1] A. Pitanti,[1,2] M. Msall,[1,3] and P. V. Santos[1,*]

[1]*Paul-Drude-Institut für Festkörperelektronik, Leibniz-Institut im Forschungsverbund Berlin e. V., Hausvogteiplatz 5-7, 10117 Berlin, Germany*

[2]*University of Pisa, Dipartimento di Fisica E. Fermi, largo Bruno Pontecorvo 3, Pisa 56127, Italy*

[3]*Department of Physics and Astronomy, Bowdoin College, Brunswick, Maine 04011, USA*

(Dated: October 10, 2025)

We present in this document additional material to support and complement the conclusions of the main manuscript.

SM1. Acoustic waves in ZnO-coated sapphire

The c-axis oriented sapphire substrate belongs to the trigonal group. The solution of the wave equation (see Eq. SM1, see below) for this material for wave propagating with wave vector **q** in the a-b crystallographic plane (i.e., in the radial (**r**) direction perpendicularly to the crystallographic c-axis) yields three types of acoustic waves:[1]

- a pure longitudinal wave polarized along the propagation direction (**r**) and phase velocity v_L;
- a pure shear wave polarized along z and phase velocity ($v_{T,z}^{-1}$);
- a pure shear wave polarized along z and phase velocity ($v_{T,\theta}^{-1}$)

In addition to these bulk modes, the substrate also supports surface acoustic modes (SAWs) confined to the surface region.

The phase velocities of these modes depend on their propagation angle with respect to the *a* crystallographic axis. The slowness surfaces (v_i^{-1}, i.e., the inverse velocity surfaces) for these waves are illustrated in Fig. SM1. The elasticity constants for crystalline sapphire used for the calculations in Fig. SM1 (as well as for crystalline ZnO and for the isotropic approximation for sapphire) are listed in Table SMI. Table SMII lists representative bulk and surface wave speeds for sapphire. While the phase velocity of the longitudinal mode is isotropic in the plane, the one for the transverse modes varies with the azymuthal angle. This angular variation in phase speed introduces anisotropies in energy flux, including concentration of the acoustic energy along caustics determined by inflections in the constant frequency surfaces in k-space, an effect known as phonon focusing [2].

Finally, the c-plane sapphire substrate supports a single surface acoustic (SAW) mode with the phase velocities v_{SAW} along the *a* and *b* axes listed in the last column of the table.

SM2. Acoustic modes of a cylindrical plane

A very instructive description of Lamb waves propagating along a fixed direction using cartesian coordinates can be found in Chapter 10 of Auld's classic book on acoustics [1]. Here, we extend Auld's one-dimensional formulation for the acoustic displacement field $\vec{u}$ of cylindrical Lamb waves propagating in a disk of thickness d_{sub}, as displayed schematically in Fig. 1(a) of the main text.

* corresponding author: santos@pdi-berlin.de

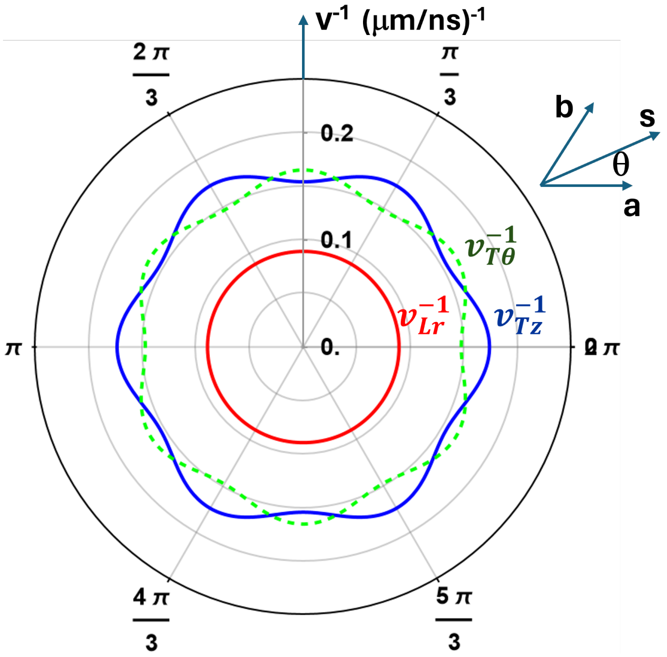

FIG. SM1. Angular dependence of the acoustic slowness for acoustic waves propagating with a wave vector $\mathbf{k_r}$ along the c plane of sapphire for longitudinal (v_{Lr}^{-1}) and transverse modes polarized along (v_{Tz}^{-1}) and perpendicularly ($v_{T\theta}^{-1}$) to the c-axis. *a* and *b* are the in-plane crystallographic axes.

TABLE SMI. Densities (ρ, in units of kg/m^3) and non-vanishing elastic constants (c_{ij}, in units of $10^{12}N/m^2$) for the materials used in this work.

Material	ρ	c_{11}	c_{12}	c_{13}	c_{33}	c_{14}	c_{44}	c_{55}	c_{56}	c_{66}
Sapphire[a]	3986	0.497	0.163	0.111	0.498	-0.024	0.147	0.148	-0.024	0.167
Sapphire (iso)[b]	3980	0.538	0.231	0.231	0.538	-	0.154	0.154	-	0.154
ZnO[3]	5665	0.2096	0.120	0.1046	0.211	-	0.0423	-	-	0.444

[a] https://www.crystran.com/optical-materials/sapphire-al2o3
[b] From COMSOL Multiphysics.

We will assume the disk material to be elastically isotropic with non-vanishing elastic constants c_{11} and $c_{12} = c_{13}$. For numerical calculations, we will use the parameters listed in Table SMII [row isotropic "sapphire (iso)"]. This approximation, on the one hand, disregards the presence of the ZnO film as well as the (quite pronounced) azymuthal dependence of the elastic properties of c-plane sapphire on the propagation angle θ (cf. Fig. 1(a), main text) in the a-b surface plane. On the other hand, it considerably simplifies the problem, enabling the derivation of analytical expressions for the field distribution. The impact of the ZnO layer will be considered as a perturbation in Sec. III B of the main text. Finally, we will only consider modes with a real propagation constant along the radial direction.

The dynamical equation for elastic deformation $\vec{u}$ can be expressed as: [1]

$$\rho \frac{\partial^2 \vec{u}}{\partial t^2} - \nabla \cdot \tau = \vec{f}, \quad \text{(SM1)}$$

where ρ is density, $\vec{u}$ is the displacement field, $\vec{f}$ the volume force, and τ the stress tensor. For the calculation of the eigenmodes, we will assume here that $\vec{f} = 0$. The wave equation is complemented by the following constitutive relations for a non-piezoelectric material[5]

TABLE SMII. Calculated longitudinal (v_L) and transverse ($v_{T,i}$) acoustic velocities for modes with polarization along i propagating along the a and b axes of a c-plane sapphire substrate. The last column summarizes the corresponding velocities of the SAW modes on a plain sapphire substrate. The calculations were carried out following the procedure delineated in Refs. 1 and 4 using the parameters for sapphire and ZnO listed in Table SMI.

Material	v_L (m/s)	$v_{T,\theta}$ (m/s)	$v_{T,z}$ (m/s)	v_{SAW} (m/s)
Sapphire a-axis	11170	6767	5742.88	5702
Sapphire b-axis	11184	6040	6473	5556
Sapphire iso	11622.7	6212.62	6212.62	5761.66

$$\tau = c\varepsilon = c\nabla_s u \tag{SM2}$$

where ε is the strain tensor, c the elastic tensor, and $\nabla_s = \frac{1}{2}(\nabla + \nabla^T)$, where ∇ is the gradient operator. In the Voigt notation, the strain vector becomes: $\varepsilon = [s_1, s_2, s_3, s_4(yz), s_4(xz), s_5(xy)]^T$.

If the propagation medium is isotropic, these modes do not depend on the propagation angle θ. When expressed in cylindrical coordinates, the problem stated by Eq. SM1 becomes 2D-dimensional in the variable space $\{r, \theta, z\}$ with the following types of solutions:

- a pure longitudinal wave polarized along the propagation direction (**r**) and phase velocity v_{Lr};
- a pure shear wave polarized along z and phase velocity (v_{Tz});
- a pure shear wave polarized along z and phase velocity ($v_{T\theta}$)

Different approaches for the solution of Eq. SM1 are reviewed by Honarvar et al. in Ref. 6. In the most general case, the acoustic field $\vec{u}(r,\theta,z,t)$ can be decomposed into three components [6] expressed in terms of scalar potential functions $\phi(r,\theta,z,t)$, $\chi(r,\theta,z,t)$, and $\psi(r,\theta,z,t)$. The component associated with $\phi(r,\theta,z,t)$ has compressional character, the other two are dilation-free (i. e., equivoluminal) shear components polarized along z [$\xi(r,\theta,z,t)$] and in the disk plane (i.e., perpendicular to z) [$\xi(r,\theta,z,t)$]. Following Honarvar *et al.*, the displacement field $\vec{u}(r,\theta,z,t)$ can be expressed as:

$$\vec{u}(r,\theta,z,t) = \nabla\phi(r,\theta,z,t) + \nabla\times(\chi(r,\theta,z,t)\hat{e}_z) + a\nabla\times(\psi(r,\theta,z,t)\hat{e}_z), \tag{SM3}$$

We search for solutions of Eq. SM3 consisting of a superposition of Bessel functions of the first kind of order n, $J_n(k_r r)$, where $n = 0,1,2,\ldots$ is the winding number. The radial wave vector k_r yields the spatial periodicity of the oscillation along $\hat{r}$ for large radial r (i.e., in the limit where the Bessel functions become essentially a cos-like function of r). We search for solutions based on trial functions of the following form:

$$\phi(r,\theta,z,t) = (B_n^+ e^{\imath k_z z})e^{\imath(-\omega t + n\theta)} J_n(k_r r) \tag{SM4}$$
$$\chi(r,\theta,z,t) = (C_n^+ e^{\imath k_z z})e^{\imath(-\omega t + n\theta)} J_n(k_r r) \tag{SM5}$$
$$\psi(r,\theta,z,t) = (D_n^+ e^{\imath k_z z})e^{\imath(-\omega t + n\theta)} J_n(k_r r). \tag{SM6}$$

Here, k_z is a wave vector component along z. The solution with $n = 0$ is the fundamental mode while the ones with $n > 0$ are helical modes with a vortex at $r = 0$. The winding number n describes the number of windings of the acoustic field during a full rotation around the z axis (i.e., as the azimuth angle θ varies from 0 to 2π), corresponding to the topological charge of the vortex.

By substituting the previous expressions into Eq. SM3, one obtains the following expression for the displacement field $\vec{u}$ in the vector basis $\{B_e^+, C_e^+, D_e^+\}$ as

$$\vec{u}(r,\theta,z,t) = \frac{1}{r} e^{i(k_z z + n\theta)} \times \tag{SM7}$$
$$\begin{pmatrix} k_r r J_{n-1}(k_r r) - n J_n(k_r r) & in J_n(k_r r) & iak_z(k_r r J_{n-1}(k_r r) - n J_n(k_r r)) \\ in J_n(k_r r) & n J_n(k_r r) - k_r r J_{n-1}(k_r r) & -ak_z n J_n(k_r r) \\ ik_z r J_n(k_r r) & 0 & ak_r r^2 J_n(k_r r) \end{pmatrix} \begin{pmatrix} B_e^+ \\ C_e^+ \\ D_e^+ \end{pmatrix}$$

With this expression for $\vec{u}(r,\theta,z,t)$, the wave equation (Eq. SM1) can be cast as an eigenvalue problem as:

$$(M_u - \rho\omega^2 I_3) \begin{pmatrix} B_e^+ \\ C_e^+ \\ D_e^+ \end{pmatrix} = 0 \tag{SM8}$$

where I_3 is the 3×3 identity matrix and

$$M_u = \begin{pmatrix} \rho\omega_L^2 & 0 & 0 \\ 0 & \rho\omega_{T\theta}^2 & 0 \\ 0 & 0 & \rho\omega_{Txz}^2 \end{pmatrix}. \tag{SM9}$$

As expected for wave propagation in an isotropic medium, the eigenvectors of M_u consist of a pure longitudinal wave polarized along the propagation direction with eigenvalue given by $\omega_L^2 = v_L^2(k_z^2 + k_r^2)$, with $v_L^2 = c_{11}/\rho$, and two degenerate transverse modes with eigenvalues $\rho\omega_{T\theta}^2 = \rho\omega_{Txz}^2 = v_T^2 k_T^2 = v_L^2(k_z^2 + k_r^2)$, with $v_T^2 = c_{44}/\rho$. v_L and v_T are, respectively, the longitudinal and transverse propagation velocities. The transverse modes have polarization in the θ and xy-planes, respectively. It is interesting to note that M_u as well as its eigenvalues $\rho\omega_i^2(k_r,k_z)$ do not depend on the winding number n, while the eigenvectors do. The corresponding eigenvectors are:

$$\hat{\mathbf{u}}_L(r,\theta,z) = \frac{1}{r} e^{i(k_L z + n\theta)} \left[+(rk_r J_{n-1}(rk_r) - nJ_n(rk_r))\,\hat{\mathbf{r}} + inJ_n(rk_r)\,\hat{\theta} + irk_L J_n(rk_r)\,\hat{\mathbf{z}} \right] \tag{SM10}$$
$$\hat{\mathbf{u}}_{T\theta}(r,\theta,z) = \frac{1}{r} e^{i(k_T z + n\theta)} \left[+inJ_n(rk_r)\,\hat{\mathbf{r}} + (nJ_n(rk_r) - rk_r J_{n-1}(rk_r))\,\hat{\theta} \right] \tag{SM11}$$
$$\hat{\mathbf{u}}_{Txz}(r,\theta,z) = \frac{1}{r} e^{i(k_T z + n\theta)} \times \tag{SM12}$$
$$\left[+iak_T(rk_r J_{n-1}(rk_r) - nJ_n(rk_r))\,\hat{\mathbf{r}} - ank_T J_n(rk_r)\,\hat{\theta} + ark_r^2 J_n(rk_r)\,\hat{\mathbf{z}} \right].$$

A. Solution for a disk of infinity radius

The eigenstates in Eqs. SM10-SM12 are the solutions of the wave equation for modes with a given frequency ω, winding number n, and (real) radial wave vector k_r propagating along the axis of a cylinder with infinity radius and length. Solutions for modes propagating with a given wave vector along the axis of an infinity long cylinder with a given radius are discussed in Ref. 6. We will consider in this section wave propagation in a disk of infinite radius but now delimited by free surfaces at $z = \pm d/2$.

In order to fullfill the boundary conditions at the disk surfaces, we will choose a basis (cf. Eqs. SM10-SM12) consisting of modes with a given wave vector components k_r along the radial direction. The solutions can be expressed as superpositions of the eigenstates expressed by Eqs. SM10-SM12 with counter-propagating wave vectors $\pm k_z$ along the z direction fullfilling the boundary conditions at the disk surfaces. The corresponding mode coefficients will be denoted as $\{B_e^\pm, C_e^\pm, D_e^\pm\}$. The boundary conditions at the free surfaces require vanishing stress (τ) components perpendicular to the boundary surfaces $z = \pm d/2$, i. e., $\tau_{zz} = \tau_{rz} = \tau_{\theta z}$. When these constraints are applied, the solutions fall into two types of modes: a pure shear wave and a wave with mixed longitudinal and shear displacements.

1. *Pure shear modes*

Purely transverse modes are associated with the scalar potential $\psi(r,\theta,z,t)$. They are polarized in the $r-\theta$ plane and propagate in the z-direction. These modes are decoupled from the modes with $r-z$ polarization (see below). Their displacement field can be written as:

$$\mathbf{u}(r,\theta,z) = iC_e\frac{e^{i\theta n}}{r}\cos\left(k_T z\right)\begin{pmatrix} -inJ_n\left(rk_r\right) \\ rJ_{n-1}\left(rk_r\right)k_r - nJ_n\left(rk_r\right)\end{pmatrix}\begin{pmatrix}\hat{r}\\ \hat{\theta}\end{pmatrix}, \tag{SM13}$$

where C_e is the mode amplitude. For $n=0$ these modes become a purely transverse mode polarized perpendicularly to the z axis. The wave vector of the modes along z, k_T, must be related to the mode angular frequency by the boundary conditions at the top and bottom surfaces of the plate according to:

$$k_z^2 = k_T^2 = \left(\frac{\omega}{v_T}\right)^2 - k_r^2 = m_T\frac{2\pi}{d}, \quad m_T = 1,2,\ldots \tag{SM14}$$

Here, $v_T = \sqrt{c_{44}/\rho}$ is the transverse acoustic velocity and the coefficient C_e the wave amplitude. These modes have an integer number of wave periods $2\pi/(k_T d)$ within the substrate of thickness d. Because the displacement component along $\hat{z}$ vanishes, so that these modes can normally not be detected using interferometry.

2. *$\hat{r}\hat{z}$-polarized modes*

The solutions for the modes polarized in the $\hat{r}-\hat{z}$ plane are a superpositions of longitudinal and transverse modes polarized in this plane with amplitudes $B_e^{\pm}$ and $D_e^{\pm}$, respectively, and wave vector components $\pm k_T$ and $\pm k_L$ along z given by

$$\begin{aligned} k_L^2 &= \left(\frac{\omega}{v_L}\right)^2 - k_r^2, \quad v_L = \sqrt{\frac{c_{11}}{\rho}} \\ k_T^2 &= \left(\frac{\omega}{v_T}\right)^2 - k_r^2, \quad v_T = \sqrt{\frac{c_{44}}{\rho}}. \end{aligned} \tag{SM15}$$

Since the disk has mirror-symmetry with respect to its central plane z=0, the solutions for the displacement field can be classified according to whether they are mirror-symmetric [even, i.e., $\mathbf{u}(r,\theta,z) = -\mathbf{u}(r,\theta,z)$] or asymmetric [uneven, i.e., $\mathbf{u}(r,\theta,z) = +\mathbf{u}(r,\theta,z)$] with respect to reflection on this plane. In both cases, they must satisfy the following determinantal equation:

$$Det = \left[\frac{\tan\left(\frac{dk_T}{2}\right)}{\tan\left(\frac{dk_L}{2}\right)}\right]^{i_s} + \frac{4c_{44}k_L k_T k_r^2}{\left(k_T^2 - k_r^2\right)\left(c_{11}k_L^2 + (c_{11} - 2c_{44})k_r^2\right)} = 0. \tag{SM16}$$

Here, the exponent $i_s = 1$ applies to symmetric modes (i.e., those for which $B_e^- = B_e^+ = B_e$ and $D_e^- = -D_e^+ = -D_e$) and $i_s = -1$ to the anti-symmetric modes ($B_e^- = -B_e^+ = -B_e$ and $D_e^- = +D_e^+ = D_e$). The corresponding equation for one-directional Lamb wave propagation can be found in Ref. 1 (Eqs. 10.18 and 10.19). The dispersion of the cylindrical Lamb modes is shown in Fig. 5 of the main text.

According to Eqs. SM15, k_L and k_T are both real for small radial wave vectors in the range $k_r < \omega/v_L$. For $\omega/v_L < k_r < \omega < v_T$, k_L becomes purely imaginary: for a thick substrate, $\tan k_L d/2 \approx\rightarrow -\imath$. The longitudinal components of the displacement associated with the compressional wave thus become localized near the boundaries. Finally, for $k_r > \omega/v_T$ k_T also becomes purely imaginary and $\tan k_T d/2 \approx\rightarrow -\imath$: the solutions in this case are surface acoustic waves (SAWs) bound to the surfaces. The extended (along z) solutions for $k_r < \omega/v_T$ consist of several dispersion branches: for $k_r > \omega/v_T$, these solutions converge to a single SAW mode per surface.

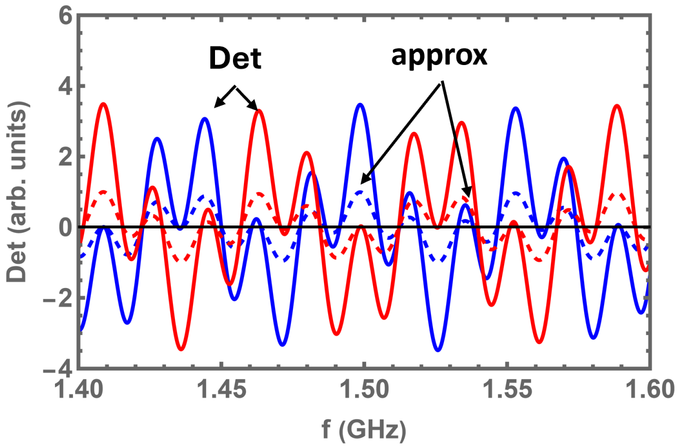

FIG. SM2. (solid line) Frequency dependence of the determinantal equation Eq. SM16 of symmetric modes (blue, $is = 1$) and anti-symmetric (red, $is = -1$). The Lamb wave solutions are those modes for which $Det = 0$. The dashed line yields the approximation given by Eq. SM19.

a. Cylindrical SAW modes: Equation 2 in the main text and the expression derived below also apply for SAWs by making the tan ratio on the left side equal to one and the replacements $k_{T,L} \rightarrow \imath\alpha_{T,L}$, where $\alpha_{T,L}$ are real numbers. The dispersion of cylindrical SAWs then becomes determined by the solutions of:

$$-4v_T^4\alpha_L\alpha_T k_{SAW}^2 + \left(\omega^2 - 2v_T^2 k_{SAW}^2\right)^2 = 0 \tag{SM17}$$

This dispersion differs from the one for SAWs propagating along a single direction by the factor of 2 within the parenthesis (the dispersion for one-dimensional SAWs is given, e.g., by Eq. 10.33 of 1). In particular, the velocity of cylindrical SAWs (listed as 5761.66 m/s in Table SMII as calculated for the isotropic approximation of c-plane sapphire, cf. Table SMI) becomes significantly lower than for 1D-SAWs (6212.62 m/s).

b. Extended Lamb modes: The solid line in Fig. SM2 displays the frequency dependence of the determinantal equation Eq. 2 for symmetric modes (i.e., $is = 1$) calculated for a sapphire substrate with a thickness $d = 425$ μm. The Lamb wave solutions are those modes for which $Det = 0$. These modes are displaced by frequencies $\Delta f_L = 2d/v_L = 13.6$ MHz corresponding to the inverse round trip of LA waves in the substrate. The solutions correspond very closely to the frequencies where the $\tan^{is}$ terms in Eq. 2 vanish, i.e., when

$$\cos\left(\frac{k_L d}{2}\right)\sin\left(\frac{k_T d}{2}\right) = 0 \tag{SM18}$$

$$\cos\left(\frac{k_R d}{2}\right)\sin\left(\frac{k_L d}{2}\right) = 0 \tag{SM19}$$

which is reproduced by the dashed line in the plot. To a very good approximation, the solutions then consist of modes satisfying simultaneously $k_i d/\pi = m_i$ for $(i = L, T)$ with integers m_i, i.e., modes for which an integer number of half cycles with length π/k_i fit within the substrate thickness d. This approximation may, however, fail at points of the dispersion where dispersion modes anti-cross (see, Fig. 5 and the accompanying discussion in the main text). Under this approximation, these solutions fall into two classes, depending on whether the disk surfaces correspond to a node or anti-node of waves with r and z polarization:

- Modes with purely shear surface displacements:

Symmetric modes with this character appear when m_L and m_T are both even, i.e., there is an even number of longitudinal and transverse half-cycle within the substrate. Equation 2 with $i_s = 1$ is then satisfied with $\sin(k_L d/2) = \sin(k_T d/2) = 0$. For the anti-symmetric modes, both m_L and m_T are non-even leading to $\cos(k_L d/2) = \cos(k_T d/2) = 0$ in Eq. 2. For both situations, the relation between the coefficients B_e and D_e is given by:

$$D_e = -\imath \frac{c_{11}k_L^2 + k_r^2(c_{11} - 2c_{44})}{2ac_{44}k_T k_r^2} B_e \tag{SM20}$$

The displacement amplitude reads:

$$\mathbf{u}(r,\theta,z) = e^{in\theta} B_e \begin{pmatrix} \frac{(k_r r J_{n-1}(k_r r) - nJ_n(k_r r))\left(2\cos(k_L z)c_{44}k_r^2 + \cos(k_T z)\left((c_{11}-2c_{44})k_r^2 + c_{11}k_L^2\right)\right)}{k_r r^2 c_{44}} \\ -\frac{J_n(k_r r)\left(2\sin(k_L z)c_{44}k_L k_T - \sin(k_T z)\left((c_{11}-2c_{44})k_r^2 + c_{11}k_L^2\right)\right)}{c_{44}k_T} \end{pmatrix} \begin{pmatrix} \hat{r} \\ \hat{z} \end{pmatrix} \tag{SM21}$$

for $i_s = 1$. The same expression, but exchanging sin $\rightarrow$ cos and cos $\rightarrow$ sin applies for the modes for $i_s = -1$. The surface displacement is restricted to the radial direction and given by:

$$\mathbf{u}\left(r,\theta,\frac{d}{2}\right) = B_e \frac{\omega^2(k_r r J_{n-1}(k_r r) - nJ_n(k_r r))}{k_r r^2 v_T{}^2} e^{i\theta n}\hat{r}. \tag{SM22}$$

Due to the absence of vertical (i.e., along z) surface displacements, these modes are not detectable by interferometry.

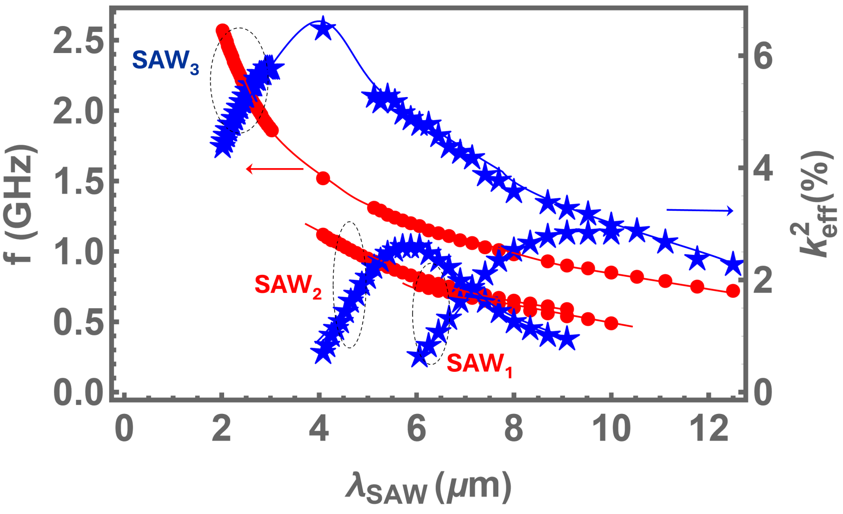

FIG. SM3. Calculated frequency (left vertical scale) and electromechanical coupling (k^2_{eff} as a function of the acoustic wavelength λ_{SAW} for piezoelectrically active SAW modes on c-plane sapphire coated with a 700 nm thick ZnO layer. The calculations were carried out using the isotropic approximation for sapphire, cf. Table SMI.

- Modes with non-vanishing vertical surface displacements

For these modes, either $i_s = 1$ and m_L and m_T uneven (leading to $\cos(k_L d/2) = \cos(k_T d/2) = 0$ in Eq. 2) or $i_s = -1$ and both m_L and m_T even ($\sin(k_L d/2) = \sin(k_T d/2) = 0$). The relation between the coefficients is given by:

$$D_e = \frac{2ik_L}{a\left(k_T^2 - k_r^2\right)} B_e \tag{SM23}$$

The displacement field depends on the symmetry of the envelope functions according to Eq. 3 of the main text, which applies for $i_s = 1$.

As in the previous case, the same expression, but exchanging sin $\to$ cos and cos $\to$ sin applies for the modes for $i_s = -1$. The radial displacement vanishes at the surface while the vertical (i.e., along z) displacement becomes:

$$\mathbf{u}\left(r, \theta, \frac{d}{2}\right) = B_e k_L \frac{\omega^2}{\omega^2 - 2k_r^2 v_T^2} J_n(k_r r) e^{in\theta} \hat{z}. \tag{SM24}$$

These modes are of particular interest due to their z-polarization near the surface, which enables detection by interferometry as well as efficient piezoelectric excitation by BAWRs. Here, one needs to consider that the strongest piezoelectric stress σ_{zz} exert by the ZnO film is mediated by the piezoelectric strain coefficient e_{33}, which couples the vertical rf-electric field in-between the BAWR electrodes to the strain along z according to $\sigma_{zz} = e_{33}\partial u_z/\partial z$.

The surface displacement amplitude for these modes given by Eq. SM24 resonates at

$$\omega = \sqrt{2} v_T k_r, \tag{SM25}$$

the so-called Lamé wave solution [1]. These modes are fully z-polarized at the surface and have a linear dispersion with a propagation velocity along the r-direction $\sqrt{2}$ higher than the transverse velocity.

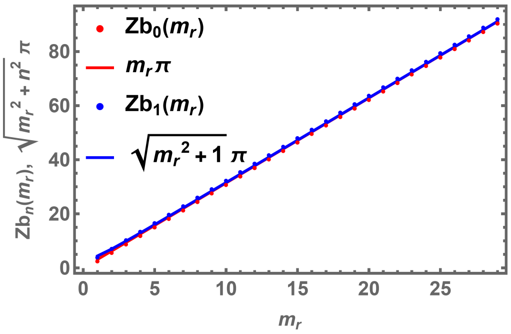

FIG. SM4. Comparison between $\sqrt{m_r^2 + n^2}\pi$ and $Z_{b_n}(m_r)$ for different winding numbers n and radial confinement indices m_r.

B. SAW modes in ZnO-coated c-plane sapphire

The presence of the textured ZnO layer, which has an acoustic velocity substantially lower than the substrate, can induce different SAW modes with phase velocities v_{SAW} and piezoelectric generation efficiency depending on the ratio between the SAW wavelength (λ_{SAW}) and the ZnO thickness.

Figure SM3 displays dependence of the SAW frequency $f_{\mathrm{SAW}} = v_{\mathrm{SAW}}/\lambda_{\mathrm{SAW}}$ and electromechanical generation efficiency k^2_{SAW} on λ_{SAW} as calculated for a c-plane sapphire substrate coated with $d_{\mathrm{film}} =$

700 nm-thick ZnO film. The calculations were carried out for a 1D model for SAW propagation using the elastic properties listed in Table SMI. Due to the much smaller acoustic velocities of ZnO as compared to sapphire, the ZnO film acts as an acoustic waveguide supporting different transverse modes denoted in the figure as SAW_i, $i = 1, 2, 3$. For simplicity, we include in the figure only modes with $k^2_{SAW} > 0.25$, which can be efficiently excited by the IDT. Note that SAW_3 has the highest k^2_{SAW} as well as the highest acoustic velocity.

C. Solution for a disk of radius r_{film}

As stated in the main text, approximate solutions for Lamb modes in a disk of finite radius r_{film} are obtained by imposing the constraint on the radial wave vector given by Eq. 7. To a very good approximation, $Z_{b_n}(m_r) \approx m_r\pi$. This assumption is confirmed by the comparison of these two quantities as a function of m_r displayed in Fig. SM4.

SM3. Radio-Frequency phase shifter

Figure SM5(a) displays the schematic diagram of a programmable rf phase shifter used for the excitation of helical waves. In the setup, the input rf drive is first split by a passive 3-fold splitter into three components. These are then shifted in phase relative to each other by two electrically controlled phase shifters. The programable attenuators and amplifiers in each of the three arms enable the control of the amplitude of the rf-drives applied to each sector BAWR. Panel (b) of Fig. SM5(b) shows a photograph of the setup.

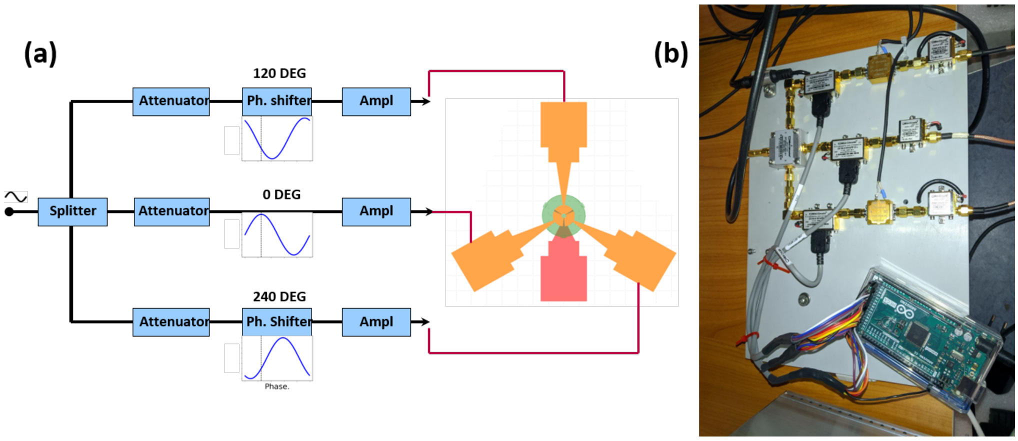

FIG. SM5. Tunable phase shifter for the excitation of helical acoustic waves. (a) The input rf-signal is split by a passive 3-fold splitter into three components, which are shifted in phase by electrically controlled phase shifters. The attenuator and amplifier allow the control of the amplitude of the rf-drives to be applied to the sector BAWRs. (b) Photograph of the setup.

SM4. Videos of helical acoustic modes

The following mp4 video files display animations of helical acoustic fields:

- Clockwise-rotating helical acoustic field (video SM-Video-CW-240208-001_F2.mp4): time evolution of a clockwise rotating field at a frequency of 1.5033 GHz (cf. Figs. 4(b)-(d) of the main text).

- Counterclockwise-rotating helical acoustic field (video SM-Video-CCW-240206-013_F2.mp4): time evolution of an counterclockwise rotating field at a frequency of 1.5033 GHz (cf. Figs. 4(f)-(h) of the main text).

The videos were elaborated from the measured phase-resolved interferometric profiles by first subtracting the unstructured background signal and then adding a fixed phase shift per frame (40 frames for a phase span of 360°).

References

[1] B. A. Auld, *Acoustic Fields and Waves in Solids* (Robert E. Krieger Publishing Company, Inc, Malabar, Florida, 1990).

[2] J. P. Wolfe, *Imaging Phonons: Acoustic Wave Propagation in Solids* (Cambridge University Press, Cambridge, 1998).

[3] K. H. Hellwege and A. M. Hellwege, *Elastic, Piezoelectric, Pyroelectric, Piezooptic, Electrooptic Constants and Nonlinear Dielectric Susceptibilities of Crystals*, Vol. 11: Elastic, Piezoelectric, Pyroelectric, Piezooptic, Electrooptic Constants and Nonlinear Dielectric Susceptibilities of Crystals (Springer Verlag, Heidelberg, 1979).

[4] M. M. de Lima, Jr. and P. V. Santos, Modulation of photonic structures by surface acoustic waves, Rep. Prog. Phys. **68**, 1639 (2005).

[5] R. Lerch, Simulation of piezoelectric devices by two- and three-dimensional finite elements, IEEE Trans. Ultrason., Ferroelectr., Freq. Control **37**, 233 (1990).

[6] F. Honarvar, E. Enjilela, A. N. Sinclair, and S. A. Mirnezami, Wave propagation in transversely isotropic cylinders, Int. J. Solids Struct. **44**, 5236 (2007).
